# Muscarinic-Dependent miR-182 and QR2 Expression Regulation in the Anterior Insula Enables Novel Taste Learning

**DOI:** 10.1523/ENEURO.0067-20.2020

**Published:** 2020-05-29

**Authors:** Nathaniel L. Gould, Alina Elkobi, Efrat Edry, Jonathan Daume, Kobi Rosenblum

**Affiliations:** 1 Sagol Department of Neuroscience, University of Haifa, Haifa 3498838, Israel; 2 Center for Gene Manipulation in the Brain, University of Haifa, Haifa 3498838, Israel

**Keywords:** mAChR, miR-182, NQO2, oxidative stress, QR2, ROS

## Abstract

In a similar manner to other learning paradigms, intact muscarinic acetylcholine receptor (mAChR) neurotransmission or protein synthesis regulation in the anterior insular cortex (aIC) is necessary for appetitive taste learning. Here we describe a parallel local molecular pathway, where GABA_A_ receptor control of mAChR activation causes upregulation of miRNA-182 and quinone reductase 2 (QR2) mRNA destabilization in the rodent aIC. Damage to long-term memory by prevention of this process, with the use of mAChR antagonist scopolamine before novel taste learning, can be rescued by local QR2 inhibition, demonstrating that QR2 acts downstream of local muscarinic activation. Furthermore, we prove for the first time the presence of endogenous QR2 cofactors in the brain, establishing QR2 as a functional reductase there. In turn, we show that QR2 activity causes the generation of reactive oxygen species, leading to modulation in Kv2.1 redox state. QR2 expression reduction therefore is a previously unaccounted mode of mAChR-mediated inflammation reduction, and thus adds QR2 to the cadre of redox modulators in the brain. The concomitant reduction in QR2 activity during memory consolidation suggests a complementary mechanism to the well established molecular processes of this phase, by which the cortex gleans important information from general sensory stimuli. This places QR2 as a promising new target to tackle neurodegenerative inflammation and the associated impediment of novel memory formation in diseases such as Alzheimer’s disease.

## Significance Statement

Most studies on molecular mechanisms underlying memory consolidation have thus far focused on the transformation of electrical and synaptic activity to post-translation modifications, mRNA translation, and gene transcription regulation. Here, we explore a less studied mechanism of the removal of an innate constraint to allow the formation of long-term memory. Our findings point to a pathway of GABA_A_ receptor control of mAChR activation, which causes the upregulation of miRNA-182, which can in turn lead to destabilization of QR2 mRNA in the rodent anterior insular cortex. The results propose a novel molecular cascade, complementary to the mRNA translation/transcription regulation underlying memory consolidation, by which the cortex gleans important information from general sensory stimuli.

## Introduction

Animals experience a continuous stream of sensory information, which is filtered by the brain according to novelty (Barry et al., 2012), inter alia. Novel stimuli merit the formation of memory and identification of these stimuli in future encounters. In the case of taste stimuli, taste memory allows the animal to identify new safe or harmful foods and accordingly to consume or avoid them, taking into account the metabolic needs and innate preferences shaped by evolution (Katz and Sadacca, 2011; Yiannakas and Rosenblum, 2017).

Neuronal filters to detect novel information can operate immediately during information acquisition or later, during memory consolidation, when short-term memories are converted to long-term ones (Alberini and Kandel, 2014; Santini et al., 2014; Lisman et al., 2018). However, the molecular mechanisms underlying such filtering processes are unclear. Theoretically, there are two major ways to create and consolidate new internal representations of sensory information in the brain. The first, more extensively studied, involves electrical and synaptic activity, post-translation modifications, mRNA translation, and gene transcription regulation (Kandel et al., 2014). The second, less studied mechanism, involves removal of an innate constraint to allow the formation of long-term memory (Genoux et al., 2002; Rappaport et al., 2015; Gai et al., 2016).

When the mammalian cortex is presented with unfamiliar input from sensory organs, neuromodulators such as acetylcholine (ACh) induce an attuned, attentive behavior, critical to notice and remember such new information (Hasselmo and McGaughy, 2004). In the case of taste stimuli, the anterior insular cortex (aIC) is the primary cortex within which reside taste representations and valence (Accolla and Carleton, 2008). Novel taste sensation induces ACh in the aIC, which enables incidental taste learning to form an internal representation of the novel taste as “safe,” a crucial nonassociative form of learning. ACh is released in many novel stimuli and memory formation modalities across the cortex and is a main target for cognitive enhancement in aged or demented people (Miranda et al., 2003; Pepeu and Giovannini, 2010). Following novel taste consumption, GABA_A_ receptors (GABA_A_Rs) enable local control of ACh release in the aIC, ensuring that only relevant gustatory events (novel taste) will cause prolonged ACh release there (Rodríguez-García and Miranda, 2016). Prolonged release of ACh in the aIC is necessary for novel taste formation and stabilization. ACh has a short-term effect on neuronal activity and excitability, however, in the aIC, it is released for many minutes and can also affect memory consolidation (Gutiérrez et al., 2003). Here, we aimed to reveal the ACh-dependent molecular and cellular events taking place hours following experiencing novel taste in the aIC and allowing the durability of a relatively weak but important form of nonassociative taste memory.

We have previously reported that novel taste memory is correlated with reduced expression of *N*-ribosyl-dihydronicotinamide quinone reductase 2 (NQO2; aka QR2), which serves as a memory constraint. However, the mechanisms upstream of QR2 expression reduction and how this reduction allows the formation of incidental taste memory are unknown. Furthermore, muscarinic ACh receptor (mAChR) activation not only leads to QR2 reduced expression, but is also known to affect reactive oxygen species (ROS) clearance (Frinchi et al., 2019). Since QR2 activity can directly influence cellular ROS levels and redox state (Cassagnes et al., 2018), it is unclear whether some of the anti-inflammatory effect of mAChR activation is mediated by QR2 suppression. If so, redox modulation via mAChR-dependent QR2 downregulation hours after novel taste consumption might affect redox-sensitive molecules within the cell, altering the neuronal intrinsic properties. As redox also plays an important role in learning and memory (Massaad and Klann, 2011) and is proposed to underlie many neurodegenerative diseases, it is therefore important to define the links among novel taste sensation, ACh, QR2, and their function in the aIC. Here, we first identify the sequential molecular events taking place in the aIC from local control of ACh release to reduced QR2 expression and suggest a possible function of QR2 in the brain.

## Materials and Methods

### Subjects

Animals used were 225–250 g male Sprague Dawley rats and 8-week-old, 20–25 g C57BL/6 (Envigo) male mice. The animals were kept in the animal core facilities at the University of Haifa, in a temperature-controlled environment (22–24°C), with a 12 h light/dark cycle (light phase, 7:00 A.M. to 7:00 P.M.). Water and rodent chow were provided *ad libitum*. All experiments were approved by the University of Haifa Animal Care and Use committee (license numbers 437/16, 487/17, 488/17, and 557/18). Animals were given 7 d of acclimatization before experimentation, and during the entire period animals were handled in accordance with University of Haifa practices and standards, in compliance with the National Institutes of Health guidelines for the ethical treatment of animals.

### Animal behavior

Animals were taught to drink water from pipettes filled with water (two 10 ml pipettes for rats and one 2 ml pipette for mice) once a day for 20 min, for 3 (rats) to 5 (mice) days. The following day, animals were given pipettes containing a novel taste (saccharin 0.1% for rats and 0.5% for mice; NaCl 0.3% for rats and 0.5% for mice). For QR2 mRNA quantification, animals were killed 3 h later. For novel taste learning in rats, long-term memory of the taste was assessed the day after the first consumption of the novel taste by a choice test between two pipettes containing water and two containing the novel taste. In mice, the day after novel taste consumption, the animals were given water in pipettes again. Then, 48 h after novel taste consumption, they were given a choice between one pipette of water and another of the novel taste. Memory, as determined by the choice test given to rats and mice, was measured by calculating a preference index for the novel taste. This was done by dividing the volume of novel taste by the total volume consumed − [novel taste/(novel taste + water)] * 100 (Rosenblum et al., 1993). Statistical analysis was performed using GraphPad Prism 7 software (GraphPad Software).

### Pharmacology

#### Materials

Pharmacological agents were diluted or dissolved in 0.9% saline, prepared by dissolving NaCl (Sigma-Aldrich) in double-distilled water (DDW). Reagents that first required dissolving in DMSO were used with vehicle containing the saline solution with DMSO diluted to a concentration equal to that of the reagent. In this manner, S29434 (synthesized by the Nancy and Stephen Grand Israel National Center for Personalized Medicine, Weizmann Institute of Science, Rehovot, Israel) 8 mg/kg in 4% DMSO and scopolamine (Sigma-Aldrich) 2 mg/kg and were applied to animals by intraperitoneal injection.

#### Microinjections

Rats were anesthetized with 0.3 ml/100 g body weight Equithesin [2.12% MgSO_4_, 10% ethanol, 39.1% 1,2-propanolol, 0.98% sodium pentobarbital, and 4.2% chloral hydrate (all from Sigma-Aldrich); Merhav and Rosenblum, 2008; Stern et al., 2013]. They were then placed in a stereotaxic device (Stoelting) and implanted bilaterally with 10 mm, 23 gauge steel guide cannulas over the IC [coordinates relative to bregma: anteroposterior (AP) 1.2 mm; mediolateral (ML) ±5.5 mm; dorsoventral (DV) 5.5 mm; Paxinos and Watson, 2007]. Cannulas were fixed with acrylic dental cement and two screws. Rats were then given a week to recover before undergoing any further experimentation.

Following the recovery period, animals began behavioral training as has been described. For microinjections 20 min before taste learning, the stylus within the guide cannulas was removed, and a 28 gauge injection cannula was inserted 0.5 mm beyond the tip of the guide cannula. This was connected to a Hamilton microsyringe via PE20 tubing, and 0.5–1 μl of vehicle or agent was injected [vehicle: 0.07 μg bicuculline, 0.07 μg muscimol (both from Tocris Bioscience), 1 μg Eserine (Sigma-Aldrich), and 13 ng S29434 in 0.08% DMSO] at 0.5 μl/min. The injection cannula was then kept for a further minute before removal to minimize retraction, prevent displacement of reagents from the injection area, and allow time for the solubilized agents to dissipate into the tissue.

### Viral vectors and infection

#### Recombinant lentiviral vector production

Self-inactivating, third-generation HIV-1-based viral vectors were produced by transient cotransfection of four endotoxin-free plasmids (Endo Free Plasmid Maxi Kit, catalog #12362, Qiagen) in Invitrogen Human Embryonic Kidney 293FT (HEK293FT; Thermo Fisher Scientific) cells (Tiscornia et al., 2006). Transfer plasmids pLenti-miR182-GFP and pLenti-control-GFP, as well as short hairpin RNA (shRNA) constructs targeting QR2 and scrambled control (Rappaport et al., 2015) were purchased from Applied Biological Materials. Cells were cultured in Invitrogen DMEM with 10% fetal bovine serum (Thermo Fisher Scientific), penicillin (100 IU/ml), and streptomycin (100 mg/ml), and were kept in a 37°C incubator with 5% CO_2_. Transfection was performed using PEI (SignaGen), according to manufacturer instructions. Medium was changed 30 min before transfection and, following a 17 h incubation, the medium was replaced with DMEM supplemented with 10% fetal bovine serum. At 48 h post-transfection, medium was harvested, cleared by low-speed centrifugation (800 RCF, 10 min, 4°C), and filtered using 0.45 μm pore filters (Nunc). Vectors were then concentrated by ultracentrifugation using the SW28 rotor (Beckman Coulter; 19,000 rpm, 2.5 h, 15°C), and the pellets obtained were finally suspended in HBSS (Sigma-Aldrich) and stored at −80°C. Vectors were titrated by transduction of HEK293FT cells using serial dilutions of the viral vector stock, along with 8 μg/μl Polybrene (Sigma-Aldrich), and GFP expression was analyzed by flow cytometry analysis 2 d later. The lentiviral titer obtained was 108 transducing units/ml.

#### Surgeries and injection

Mice were anesthetized with ketamine and Domitor (0.5 mg/kg, i.p., each) and were given Norocarp 0.5 mg/kg (i.p.). After 40 min, the mice were placed in a robotic stereotaxic device (NeuroStar). The scalp was cut centrally along the length of the skull, to expose the bregma and λ. Using bregma and λ as reference points for the drilling and injection site (coordinates for aIC relative to bregma: AP 0.86 mm, ML ±3.4 mm, DV 4 mm), 0.8 μl of virus was injected at a rate of 0.05 μl/min using StereoDrive and InjectoMate software (NeuroStar). Before injection, the syringe (Hamilton) was kept motionless in the injection site for 5 min. Following the injection, the syringe was left motionless in the injection site for a further 10 min to minimize retraction. Animals were given a week to recover, and a further week to a month to ensure full viral expression before any further experimentation.

#### Brain dissection and tissue preparation

Mice were killed by cervical dislocation, and brains were immediately removed and flash frozen on a tin foil platform floated on liquid nitrogen. Brains were kept in −80°C, and eventually were transferred to a Leica CM 1950 Cryostat and equilibrated to −15°C. For quantitative PCR, samples were dissected from a 1-mm-thick coronal slice (starting at bregma: AP, 1.18 mm; ML, ±3 mm; DV, 3.6 mm; ending at bregma: AP, 0.14 mm; ML, ±3.6 mm; DV, 4.1 mm; Franklin and Paxinos, 2008) using a tissue-punching device. Rats were killed by guillotine, brains were immediately removed, and the IC was dissected directly, using the medial cerebral artery and rhinal fissure as place markers. With both animals and dissection methods, samples were finally placed in 1.5 ml tubes and kept at 80°C until further processing. For the isolation of small molecules from mouse brain cytoplasm, brains were immediately placed on ice following sacrifice. The cortices were removed and mechanically homogenized with a teflon pestle homogenizer, in 1 ml reaction buffer (50 mM Tris, 150 mM NaCl, adjusted to pH 8.5 with HCl). The lysed samples were then centrifuged for 10 min at 1,000 RCF, and the supernatant was placed into Amicon Ultra 2 ml Ultracel 3K (Merck) centrifugal filters. Small molecule filtrate was then collected following a 1 h centrifugation of the samples through the centrifugal filters at 4,000 RCF in a swinging bucket rotor centrifuge. 

#### Western blot

Kv2.1 oligomerization was measured as previously described in the study by Cotella et al. (2012). Tissue samples were homogenized in nonreducing lysis buffer (HEPES 10 mm, EGTA 2 mm, EDTA 2 mm, 1× phosphatase inhibitor cocktail 3, and 1× protease inhibitor cocktail; all from Sigma-Aldrich). Protein content was measured using the BCA Protein Assay Kit (GE Healthcare). Samples were diluted in 2× sample buffer containing 4% SDS to 1 μg protein/1 μl. No β-mercaptoethanol was added to samples used to measure cysteine oxidation-dependent clumping. Samples were briefly vortexed, and 10 μl was loaded in precast 4–20% Mini-PROTEAN TGX Stain Free Gels (Bio-Rad). Samples were transferred to PVDF membranes, washed three times in TBST, blocked for 2 h in blocking buffer (Bio-Rad), and exposed to a mouse monoclonal KV2.1 antibody (1:500; UC Davis/NIH NeuroMab) overnight. After three further washes in TBST, membranes were incubated with anti-mouse HRP-conjugated secondary antibodies at room temperature for 1 h (1:10,000; Millipore Bioscience Research Reagents) and washed a further three times. Immunoblotting was conducted using the Westar Supernova Kit (Cyanagen), images were acquired with a CCD camera, and analysis was done using Quantity One software (Bio-Rad). KV2.1 clumping was calculated by the ratio of oligomeric (∼200 kDa) to monomeric (∼100 kDa) bands seen, as previously described (Cotella et al., 2012). Statistical analysis was performed using Graph Pad Prism 7 software.

### RNA extraction, reverse transcription, and qPCR

#### RNA extraction

TRI Reagent (300 μl/1 mm^3^ sample) was added directly to excised brain tissue, on ice. Samples were homogenized and transferred to 1.5 ml tubes, and 1-bromo-3-chloropropane was then added at one-tenth of the volume of TRI Reagent and mixed thoroughly. Following phase separation by chilled (4°C) centrifugation for 15 min at 12,000 RCF, 2-propanol was added in equal volume to the RNA phase taken, in new tubes. After a 30 min chilled centrifugation at 12,000 RCF, the pelleted RNA was washed with 300 μl ice-cold 75% ethanol. A final chilled centrifugation for 10 min at 7600 RCF followed, and the resultant pellet was air dried and eluted in 30 μl of ultra-pure water (Biological Industries). All reagents used were purchased from Sigma-Aldrich, unless otherwise stated.

#### Reverse transcription and qPCR

RNA was reverse transcribed to cDNA using the Applied Biosystems (Thermo Fisher Scientific) High Capacity cDNA Reverse Transcription (RT) kit. To obtain QR2 pre-mRNA, the RT kit random primers were replaced with a QR2 Exon 6/Intron 6 targeting primer (primer sequence: CATTTAATCGAGAGGAAAGG), as well as a reference HPRT mRNA primer (exon 8 targeting primer sequence: CCCTGAAGTACTCATTATAG). Before RT with these specific primers, the RNA was treated with DNase I (AMPD1-1KT, Sigma-Aldrich) to prevent nonspecific Serpin 6a amplification from the QR2 primer during qPCR. To verify that no genomic QR2 or HPRT was being measured, one sample from each group underwent RT without reverse transcriptase. The resultant cDNA was then used in TaqMan (Thermo Fisher Scientific) gene expression assays.

TaqMan primers used were QR2 (human, Hs01056948_m1; mouse, Mm01332867_m1; and rat, Rn01434728_g1), which were normalized to GAPDH (mouse, Mm99999915_g1; human, Hs99999905_m1; and rat, Rn99999916_s1) or HPRT (mouse, Mm00446968_m1). For miRNA-182 (miR-182), TaqMan MicroRNA kit was used (002599) and was quantified against U6 (001973). Relative quantitation was performed by calculating ΔΔCt of the target genes. Statistical analysis was performed using GraphPad Prism 7 software.

### Activity assays

#### Reverse-phase HPLC profile of QR2 activity assay

QR2-dependent production of menadiol was measured as previously described (Boutin et al., 2005), with modifications. Cytoplasmic small molecules (CSMs), collected as described above, were applied to an Acquity Ultra High Performance LC (Waters), and 10 μl of the samples were injected into a Spherisorb 5 mm C8 Analytical Column (Waters). The elution system consisted of a linear 30–85% acetonitrile gradient in 0.1% aqueous formic acid over 9 min (flow rate, 1 ml/min), while absorption at 280 nm was measured. Menadione 1 mm either alone or with QR2 500 nm (both from Sigma-Aldrich) was added to the CSMs, and was similarly run. An increase in menadiol product formation was measured every 10 min at the 8.5 min mark of each of five injections. S29434 was added in increasing doses (62.5, 125, 250, and 500 nm), and changes in the rate and amplitude of menadiol formation were measured. Menadiol concentrations were measured by the area under the curve of peak formation, using Empower 2 software (Waters).

#### QR2 and NQO1 activity assay

QR2 or NQO1 (Sigma-Aldrich) assays were conducted in 50 mm Tris, 150 mm NaCl buffer adjusted to pH 8.5 with HCl. To 100 μl reactions in a black-walled, clear bottom, 96-well plate (Thermo Fisher Scientific), QR2 or NQO1 enzyme (10 nm) was added, along with both BNAH (1-benzyl-1,4-dihydronicotinamide; Tocris Bioscience) and menadione 100 μm. The fluorescence of BNAH was monitored every 30 s by excitation with 340 nm and emission at 440 nm using a Tecan Infinite M200 PRO fluorescence microplate reader. To validate inhibitor efficacy and enzymatic activity, either S29434 (200 nm) or dicoumarol (200 μm) was added to the reaction. Each condition was done with or without enzyme, the initial linear phase of enzymatic activity was measured, and the nonenzymatic BNAH signal decay was subtracted from each relevant enzymatic reaction.

#### Brain lysate quinone reductase activity assay

Brains were briefly removed from 3- to 4-month-old C57BL/6 mice and placed on ice. The cortices were dissected and homogenized using a glass-Teflon homogenizer in 5 μl/mg tissue with 50 mm Tris and 150 mm NaCl buffer adjusted to pH 8.5 with HCl and containing 1 mm *n*-octyl-B-d-glucopyranoside. Samples were then centrifuged at 4°C for 5 min at 12,000 RCF, and the supernatant was kept. Each sample used for the activity assay was loaded into a 96-well, clear flat-bottomed plate (Thermo Fisher Scientific) at a final volume of 100 μl, with 100 μm BNAH either with or without 200 nm of S29434. The fluorescence of BNAH was monitored every 30 s by excitation with 340 nm and emission at 440 nm using a Tecan Infinite M200 PRO fluorescence microplate reader, and the initial linear phase of the reaction was measured.

#### Cell-based ROS assay

HEK293FT cells were cultured in Invitrogen DMEM (Thermo Fisher Scientific) supplemented with fetal bovine serum (final concentration, 10%), penicillin-streptomycin antibiotics, and l-glutamine. Cells were grown in 100 mm plates until 80–90% confluence and were passaged every 2–3 d. For cellular ROS detection, cells were collected, centrifuged, and resuspended in fresh Minimum Essential Medium without phenol red. A total of 50,000 cells/well were seeded on poly-l-lysine-coated Nunc black, 96-well, clear flat-bottomed plates (Thermo Fisher Scientific) and left to adhere for 3 h.

The DCFDA (2′,7′-dichlorofluorescin diacetate) ROS detection kit (Abcam) protocol was then followed. Briefly, cells were incubated with 20 μm DCFDA for 45 min in the dark at 37°C, and then treated with DMSO, S29434 and ascorbic acid. The QR2 inhibitor S29434 was dissolved in DMSO to 20 mm and Ascorbic Acid (Sigma-Aldrich) was solubilized in DDW to 55 mm. Both were subsequently diluted using the provided buffer from the Abcam kit (S29434 and ascorbic acid final concentrations, 20 μm and 5 mm, respectively), supplemented with 10% fetal bovine serum. In all treatments, DMSO was present in equal concentration of 0.1%. The cells were incubated for a further 4 h with the different treatments in the dark at 37°C. ROS was measured using a Tecan Infinite M200 PRO fluorescence microplate reader, with excitation/emission of 485/535 nm. Statistical analysis was performed using GraphPad Prism 7 software.

### Statistical analysis

Data collected were tested for normality using Shapiro–Wilk and Kolmogorov–Smirnov normality tests, when not transformed/normalized. All normally distributed data were analyzed using Student’s *t* test, one-way ANOVA, two-way ANOVA, or two-way repeated-measures ANOVA, followed by Tukey’s *post hoc* analysis. For data not normally distributed, or data that first required normalization before analysis (e.g., when combining separate sets of experimental data for Western blot analysis), nonparametric Mann–Whitney test, or Kruskal–Wallis test followed by Dunn’s multiple-comparisons test were used. All data are presented as the mean ± SEM. All descriptive statistics, normality tests, and parametric and nonparametric tests were conducted using GraphPad Prism 7 (GraphPad Software) and SPSS 25 (IBM) software.

### Data availability

All statistical analyses are available in Extended Data Table 1: Detailed Statistical Analysis.

10.1523/ENEURO.0067-20.2020.t1-1 Table 1-1 Detailed Statistical Analysis. Download Table 1-1, DOCX file.

## Results

### QR2 inhibition in the aIC rescues scopolamine-induced amnesia

**Figure 1.**
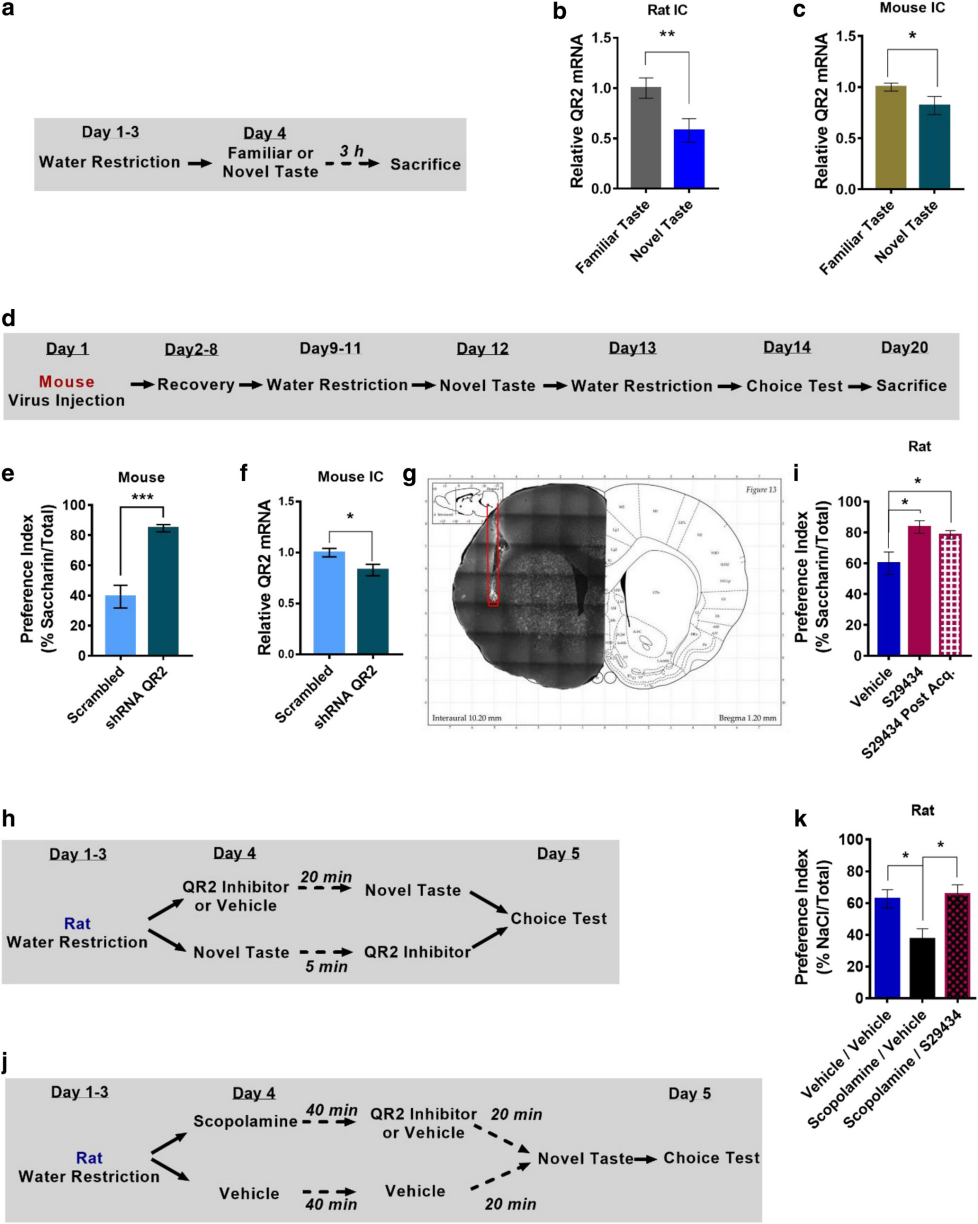
QR2 inhibition in the aIC rescues scopolamine-induced amnesia. ***a***, Rats were given a novel or familiar taste and killed 3 h later. ***b***, A significant reduction in QR2 mRNA was measured in the aIC of rats following novel taste. ***c***, QR2 mRNA expression was significantly reduced in the aIC of mice following novel taste. ***d***, Mice injected with shQR2 or scrambled lentivirus to the aIC underwent novel taste learning, and 2 d later they were given a choice test and preference was assessed. ***e***, Mice injected to the aIC with shQR2 showed significantly improved novel taste memory compared with controls. ***f***, Mice injected to the aIC with shQR2 showed significantly reduced QR2 mRNA levels compared with controls. ***g***, Cannula position validation following behavioral tests. ***h***, Rats were given a novel taste after receiving an intracranial infusion of vehicle or S29434 20 min prior, or received a novel taste and then received S29434 5 min later. The next day, the preference for the novel taste was measured. ***i***, S29434-mediated inhibition of QR2 significantly improved novel taste memory, whether given before or after novel taste presentation. ***j***, Rats were given a novel taste after having first received an intracranial infusion of vehicle or S29434 20 min prior and an intraperitoneal injection of vehicle or scopolamine 1 h before. The following day taste memory was tested. ***k***, Scopolamine significantly impedes novel taste memory, while S29434 prevents scopolamine-induced memory deficit. Data are shown as the mean ± SEM. *p < 0.05; **p < 0.01; ***p < 0.001. See supporting data in Extended Data Figure 1-1 and Extended Data Figure 1-2.

We have previously shown that novel taste consumption reduces mRNA levels of QR2 in the rat aIC (Rappaport et al., 2015). To repeat and expand this finding to other species, we extended our research to mice, where more genetic tools are readily available. We therefore measured mRNA levels of QR2 in the aIC in both rats and mice following novel taste consumption (Fig. 1*a*). In agreement with Rappaport et al. (2015), 3 h following novel taste consumption QR2 mRNA was significantly reduced in the aIC of both rats and mice (Fig. 1*b*,*c*), but not in other non-taste-related brain regions (Extended Data Fig. 1-1). To test whether, as in rats, reducing QR2 expression in the mouse aIC would also result in improved novel taste memory, a lentivirus harboring shRNA targeting QR2 (shQR2) was made, as well as a scrambled control (Extended Data Fig. 1-2). Following stereotaxic injection of the virus into the aIC, mice were given a week to recover and then were taught to drink water from pipettes. The mice were then given saccharin (0.5%) and, on the following day, water once again. A day later, the mice were given both water and saccharin in pipettes, and preference for the novel taste (saccharin) was measured (Fig. 1*d*). Mice expressing shQR2 drank significantly more saccharin than the animals injected with a scrambled control (Fig. 1*e*) and showed significantly reduced levels of QR2 mRNA (Fig. 1*f*) in aIC only (Extended Data Fig. 1-2). Hence, in mice, as in rats, reduction of QR2 expression in the aIC results in improved novel taste memory.

10.1523/ENEURO.0067-20.2020.f1-1 Figure 1-1 QR2 mRNA expression is unchanged in brain areas not associated with taste memory following novel taste consumption. ***a***, QR2 mRNA is unchanged in rat OC following novel taste consumption. ***b***, QR2 mRNA is unchanged in mouse CA1 following novel taste consumption. Download Figure 1-1, TIF file.

**Figure 2.**
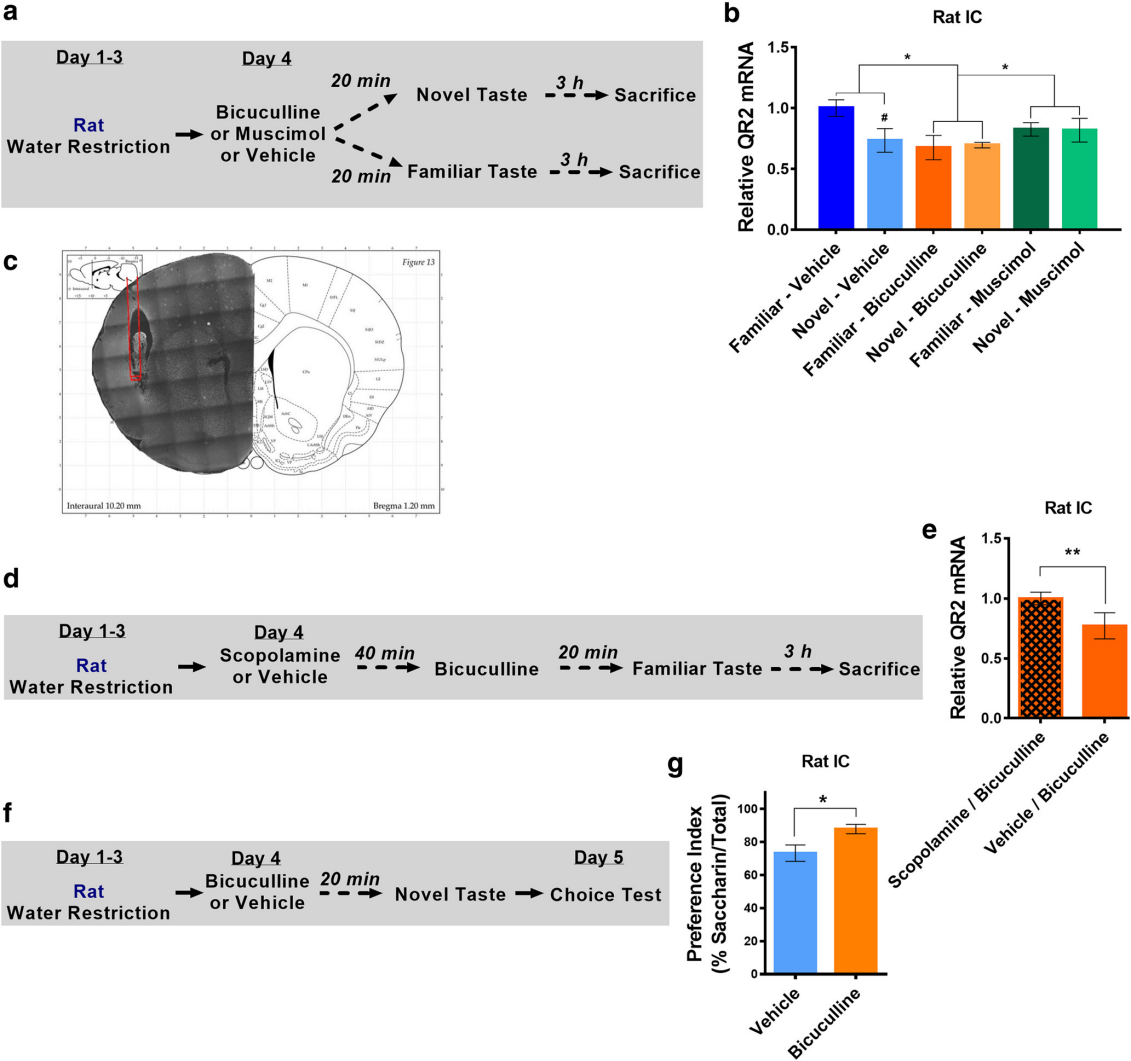
Local GABA_A_ receptor activity in the aIC is upstream of muscarinic AChR-dependent reduction in QR2 mRNA levels. ***a***, Rats were given a novel or familiar taste 20 min after having received injections of vehicle, bicuculline, or muscimol in the aIC and were killed 3 h later. ***b***, A significant reduction of QR2 mRNA was measured in the aIC of rats that received injections of a novel taste and vehicle in the aIC. Animals that received bicuculline all showed a reduction in QR2 mRNA, while no reduction in QR2 mRNA was measured in animals receiving muscimol. *Main effect (treatment); #effect within treatments. ***c***, Implanted cannula positions were validated following behavioral tests. ***d***, Rats received either scopolamine or vehicle and 40 min later received local aIC infusion of bicuculline. After 20 min, the animals were given a familiar taste and were killed 3 h later. ***e***, Scopolamine prevents the local, bicuculline-dependent reduction in QR2 mRNA in rat aIC. ***f***, Rats received injections of either bicuculline or vehicle locally to aIC, and 20 min later were given a novel taste. The next day, the animals were given a choice test between the novel taste and water, and preference for the newly learned taste was measured. ***g***, Rats that received bicuculline had significantly better memory of the novel taste compared with controls. Data are shown as the mean ± SEM. #*p* < 0.05; *p < 0.05; **p < 0.01. See supporting data in Extended Data Figure 2-1.

10.1523/ENEURO.0067-20.2020.f1-2 Figure 1-2 Lentivirus containing either shRNA targeting QR2 or a scrambled control injected to aIC did not alter QR2 mRNA expression in CA1. ***a***, Diagram of lentivirus containing shRNA targeting QR2, or a scrambled control, used to reduce QR2 expression in mice. ***b***, QR2 expression in CA1 remains unaffected by local aIC infection with lentivirus harboring shRNA targeting QR2. Download Figure 1-2, TIF file.

Functionally, systemic inhibition of QR2 with a small-molecule inhibitor (S29434) results in enhanced novel taste memory. To test whether this effect of external reduction of QR2 activity is dependent specifically on the aIC, we stereotaxically injected S29434 (13 ng; see Materials and Methods) or vehicle into the rat aIC (Fig. 1*g*) either 20 min before novel taste consumption (0.1% saccharin) or 5 min afterward, and tested taste preference the following day (Fig. 1*h*). Since rats share the same QR2 effects with mice, we opted for the larger rodent to conduct implantations to reduce relative damage to the brain and to ease correct cannula insertion (by virtue of a larger aIC). Rats injected with S29434 either before or after novel taste consumption demonstrated significantly greater consumption of saccharin a day later, indicating a stronger memory of the novel and safe taste experienced the previous day (Fig. 1*i*). Moreover, the enhanced incidental taste learning after inhibition of QR2 and the reduction in QR2 mRNA levels hours following novel taste experience are indicative of QR2 involvement in memory consolidation.

Reduced expression of QR2 in the aIC following the consumption of novel taste is mAChR dependent (Rappaport et al., 2015). To test whether the reduction in QR2 mRNA levels is downstream of the effect of mAChR on novel taste memory, we injected rats with either the mAChR antagonist scopolamine (2 mg/kg, i.p.) or vehicle 40 min before stereotaxic administration of S29434 or vehicle in the aIC. After 20 min, the animals were subjected to the novel taste learning paradigm (Fig. 1*j*). Since taste novelty, but not the taste itself, was shown to affect QR2 in the study by Rappaport et al. (2015), NaCl (0.3%) was used instead of saccharin to assess the generalizability of the effect of novelty on QR2 across different tastes. Scopolamine significantly reduced novel taste memory by 25% compared with controls, while S29434 rescued this amnesic phenotype (Fig. 1*k*). These results suggest that QR2 is reduced across species in the rodent aIC and is a new addition to the currently known downstream targets of mAChR activation during novel taste memory formation.

### Local GABA_A_ receptor activity in the aIC is upstream of muscarinic AChR-dependent reduction in QR2 mRNA levels following novel taste experience

QR2 expression reduction following novel taste consumption is found only locally, within the aIC. We therefore aimed to establish how QR2 expression reduction is contained only within the relevant brain area necessary for the long-term storage of taste memory. GABA_A_Rs tonically control local ACh release in the aIC from distal nucleus basalis magnocellularis cholinergic neurons, enabling ACh influx into the aIC during novel, but not familiar, taste consumption (Rodríguez-García and Miranda, 2016). We hypothesized that the mAChR-dependent reduction in QR2 mRNA levels in the aIC following novel taste consumption is regulated by the local GABA_A_R disinhibitory circuit. To test this hypothesis, we stereotaxically injected bicuculline (GABA_A_R antagonist, 0.07 μg), muscimol (GABA_A_R agonist, 0.07 μg), or vehicle into the rat aIC 20 min before consumption of either a familiar or novel taste, and the rats were killed 3 h later (saccharin; Fig. 2*a*). Consumption of a novel taste reduced QR2 mRNA levels compared with a familiar taste (water) in the control groups receiving vehicle (Fig. 2*b*). However, bicuculline caused a reduction in QR2 expression regardless of taste novelty/familiarity, while muscimol prevented the reduction in QR2 mRNA levels following novel taste consumption. Notably, however, muscimol did hold QR2 expression in an intermediate level, nonsignificantly reduced compared with controls. No change in QR2 expression was measured in a control cortex, the occipital cortex (OC), of these animals (Extended Data Fig. 2-1).

10.1523/ENEURO.0067-20.2020.f2-1 Figure 2-1 Local aIC GABA_A_ receptor antagonism and scopolamine injections do not affect QR2 mRNA in the occipital cortex. ***a***, QR2 mRNA expression is unchanged following novel taste or antagonism of GABA_A_R with bicuculline locally in the aIC. ***b***, QR2 expression remains unchanged in the OC of rats, following local antagonism of GABA_A_R in the aIC, with or without prior injections of scopolamine. Download Figure 2-1, TIF file.

To verify that bicuculline induced reduction in QR2 mRNA levels following familiar taste consumption was due to subsequent mAChR activation, rats were given scopolamine or vehicle 40 min before stereotaxic administration of bicuculline into the aIC (Fig. 2*c*,*d*). Scopolamine injection before bicuculline microinfusion prevented the GABA_A_R-dependent reduction in QR2 mRNA expression in the aIC (Fig. 2*e*). The OC did not show any change in QR2 expression (Extended Data Fig. 2-1). Next, we hypothesized that stereotaxic administration of bicuculline into the aIC would also improve novel taste memory. To test this hypothesis, rats were stereotaxically injected with bicuculline or vehicle into the aIC and were given saccharin 20 min later. The following day, novel taste memory was measured by preference index (Fig. 2*f*). The group that received bicuculline showed enhanced memory, having a significantly increased preference for saccharin (Fig. 2*g*). These results suggest that a local GABA_A_R-dependent release of ACh in the aIC following novel taste consumption activates mAChR, which causes a reduction in QR2 and allows the formation of incidental taste memory.

### miR-182 is upregulated locally in the aIC following novel taste consumption in a GABA_A_R-mediated, muscarinic AChR-dependent manner

**Figure 3.**
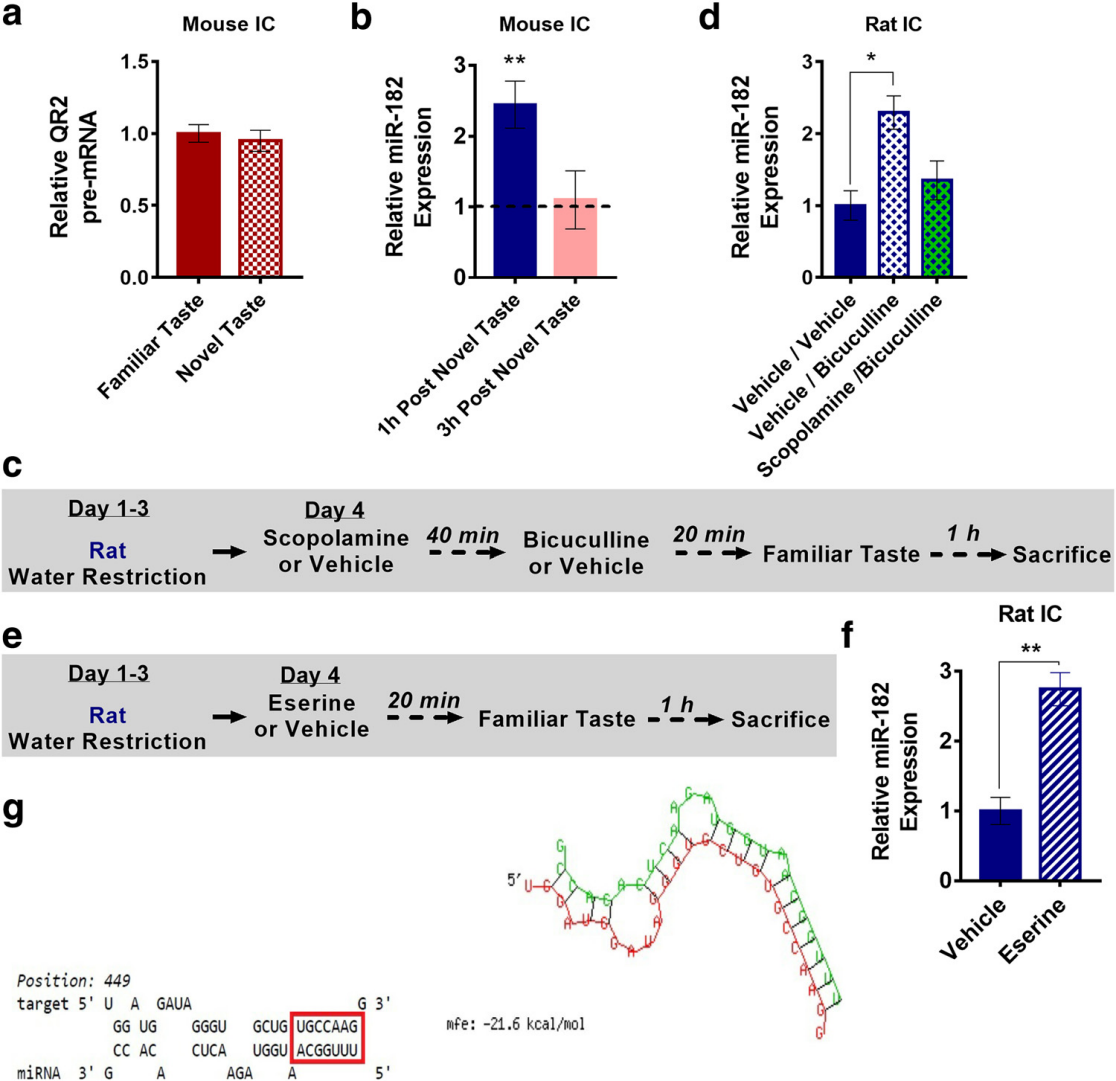
miR-182 is upregulated locally in the aIC following novel taste consumption in a GABA_A_R-mediated, muscarinic AChR-dependent manner. ***a***, QR2 pre-mRNA levels were unchanged in the aIC of mice following novel taste. ***b***, miR-182 expression was measured 1 and 3 h following familiar or novel taste consumption in the aIC of mice. miR-182 expression was elevated 1 h, but not 3 h, following novel taste. ***c***, Rats were given intraperitoneal injections of scopolamine or vehicle 1 h—and then aIC infusions of vehicle or bicuculline 20 min—before familiar taste. Animals were killed 1 h later, and miR-182 was measured. ***d***, miR-182 expression was significantly increased in the aIC after 1 h, in rats that received bicuculline locally to the aIC and vehicle intraperitoneally. ***e***, Infusions of vehicle or Eserine then were given locally to the aIC of rats 20 min before familiar taste. Animals were killed 1 h later, and miR-182 was measured. ***f***, miR-182 expression significantly increased in rat aICs that received Eserine locally, compared with vehicle. ***g***, One of four predicted miR-182 seeds to QR2 mRNA binding sites (miR-182, green; QR2 mRNA, red) in the rat. Data are shown as the mean ± SEM. *p < 0.05; **p < 0.01. See supporting data in Extended Data Figure 3-1 and Extended Data Figure 3-2.

Since QR2 pre-mRNA was not altered following novel taste consumption (Fig. 3*a*), an open question remained as to how novelty reduces QR2 mRNA in the aIC. One possible mechanism is the destabilization of QR2 mRNA by miR. Some miRs have been shown to play an important role in memory formation (Woldemichael and Mansuy, 2016); therefore, we selected 15 prime miR candidates and measured their expression directly 1 and 3 h following novel or familiar taste consumption. Of these, miR-182 was elevated 1 h following novel taste consumption in the aIC of mice (Fig. 3*b*).

10.1523/ENEURO.0067-20.2020.f3-1 Figure 3-1 miR-182 expression does not increase in the mouse CA1 following novel taste consumption or in the rat OC following pharmacological manipulation locally to the rat aIC. ***a***, miR-182 levels remain unaltered in the CA1 of mice following novel taste consumption. ***b***, miR-182 levels remain unchanged in the OC of rats, following local aIC antagonism of GABA_A_R with bicuculline, with or without prior injection of scopolamine. Download Figure 3-1, TIF file.

10.1523/ENEURO.0067-20.2020.f3-2 Figure 3-2 Predicted hybridization sites of miR-182 to QR2 mRNA in the human, mouse, and rat genome. Download Figure 3-2, TIF file.

We then aimed to see whether its induced expression following novel versus familiar taste consumption is controlled by upstream neurotransmitters (i.e., GABA_A_R-mediated release of ACh and mAChR activation), similar to QR2 (Fig. 2), as seen in rats. To do so, rats were injected intraperitoneally with either scopolamine or vehicle and were stereotaxically injected with bicuculline or vehicle into the aIC. The rats then received a familiar taste and were killed 1 h later (Fig. 3*c*). Animals that received bicuculline and vehicle showed a significant increase in miR-182 in the aIC compared with those that received the vehicle alone. However, scopolamine prevented this increase and kept miR-182 levels similar to those seen in control animals (Fig. 3*d*), suggesting that the increase in miR-182 levels is mediated by mAChR activation in the aIC alone (Extended Data Fig. 3-1).

To assess whether local, aIC-specific activation of mAChR would cause an increase in miR-182 levels (a mirror effect of scopolamine), the experiment was repeated with local infusion of Eserine, an ACh esterase inhibitor, which prolongs the innate effect of ACh. Namely, animals that were taught to drink from pipettes were then injected intracranially either with Eserine (1 μg/hemisphere) or vehicle 20 min before familiar taste presentation, and miR-182 was measured 1 h following familiar taste consumption (Fig. 3*e*). Animals that received Eserine showed a significant increase in miR-182 levels, similar to those receiving bicuculline or a novel taste (Fig. 3*f*). With such closely tied paths for QR2 and miR-182 within the aIC following novel taste consumption, GABA_A_R modulation, and mAChR activation, we next examined whether miR-182 hybridization to QR2 mRNA is predicted *in silico*. Using BiBiServ2 (Rehmsmeier et al., 2004), a number of miR-182 seed-to-QR2 mRNA hybridizations were identified in the mouse, rat, and human (Fig. 3*g*, Extended Data Fig. 3-2), placing miR-182 as a leading candidate for QR2 expression reduction following novel taste experience.

### Increased miR-182 expression in the aIC reduces local QR2 mRNA levels and improves novel taste learning

**Figure 4.**
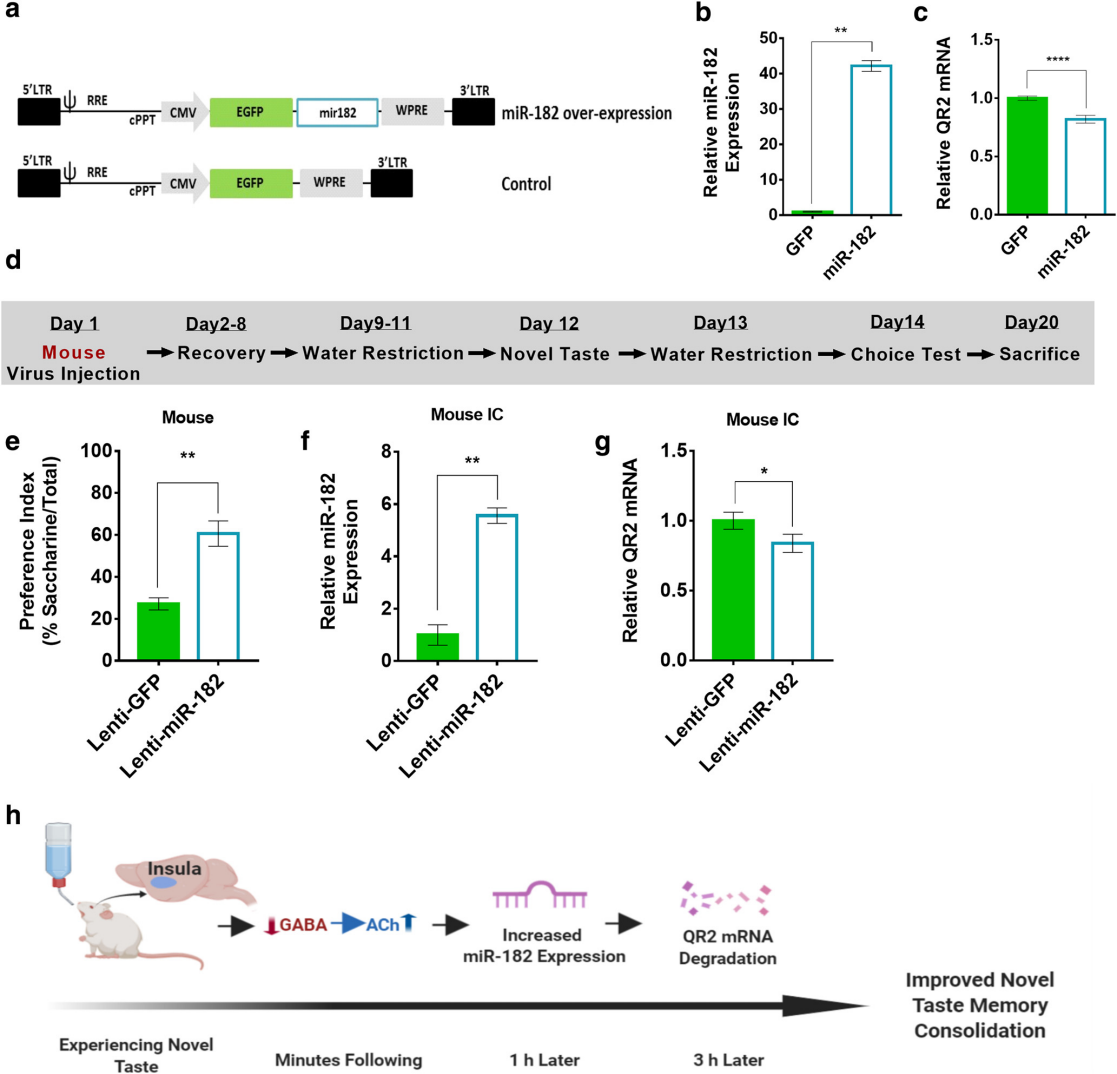
Increased miR-182 expression in the aIC reduces local QR2 mRNA levels and improves novel taste learning. ***a***, Lentiviral vectors containing either reporter GFP and miR-182, or GFP alone, under the CMV promoter were generated. ***b***, Transfected HEK293FT cells showed significantly increased levels of miR-182 with the overexpression plasmid, compared with that of GFP. ***c***, QR2 mRNA measured showed significantly reduced expression in cells transfected with the miR-182 plasmid. ***d***, Mice were injected with an miR-182 or GFP construct harboring lentivirus in the aIC and then underwent novel taste learning, and were then given a choice test to assess taste memory. Mice were killed to assess infection efficacy. ***e***, Mice injected with miR-182 showed significantly improved memory for the novel taste compared with GFP-injected controls. ***f***, aIC samples taken from miR-182 overexpression vector-injected mice following completion of behavioral experiments show a significant increase in miR-182 expression compared with GFP-injected controls. ***g***, QR2 mRNA was significantly reduced in the aIC of mice injected with the miR-182 virus, compared with controls. ***h***, Schematic representation of the train of events on novel taste exposure, leading to reduced QR2 expression and improved novel taste memory in the rodent aIC. Data are shown as the mean ± SEM. *p < 0.05; **p < 0.01; ***p < 0.001; ****p < 0.0001. See supporting data in Extended Data Figure 4-1.

To test whether miR-182 can cause reduction in QR2 mRNA levels and enhance novel taste learning, we synthesized constructs encoding miR-182 overexpression and GFP reporter or GFP reporter alone (Fig. 4*a*). First, we tested the effect of the constructs by transfection of HEK293FT cells, which are known to express QR2 (Geiger et al., 2012), and miR-182 and QR2 expression levels were measured (Fig. 4*b*,*c*). HEK293FT cells treated with miR-182-encoding plasmids showed a significant reduction in QR2 mRNA expression levels compared with GFP controls.

10.1523/ENEURO.0067-20.2020.f4-1 Figure 4-1 Mice injected in IC with a lentivirus harboring shRNA targeting QR2 do not show changes in QR2 mRNA or miR-182 expression in the hippocampus CA1. ***a***, miR-182 levels in CA1 were not elevated following local aIC infection with a lentivirus overexpressing miR-182. ***b***, QR2 mRNA levels in CA1 were not changed following local aIC infection with a lentivirus overexpressing miR-182. Download Figure 4-1, TIF file.

To assess the effect of miR-182 overexpression on novel taste memory and the ability to cause a reduction in QR2 mRNA expression levels in the brain, mice were stereotaxically injected into the aIC with a lentivirus containing the miR-182 or GFP control constructs under the CMV promoter. The mice were then trained to drink from pipettes and were given a novel taste, and 2 d later they were given a choice test to measure the strength of the novel taste memory (Fig. 4*d*). The group injected with miR-182 showed significantly improved taste memory compared with controls, as indicated by the increased preference for the newly learned taste (Fig. 4*e*). Validation of the virus delivery and infection was done by measuring miR-182 expression levels a week later. The miR-182 group showed significantly elevated miR-182 expression levels in the aIC compared with controls (Fig. 4*f*), elsewhere, not previously (hippocampus; Extended Data Fig. 4-1), which caused a significant reduction in QR2 mRNA in the aIC only (Fig. 4*g*). Thus, the expression of miR-182 affects QR2 mRNA expression in the aIC and improves novel taste memory. Overall, as described in Figure 4*h*, novel taste stimuli cause reduced GABA_A_R activation, which leads to local and prolonged influx of ACh and activation of mAChR. In turn, miR-182 expression is increased, eventually leading to reduced QR2 expression, among its other known targets, thus improving the long-term persistence of the novel taste memory.

### Endogenous QR2 activity generates cellular ROS and affects Kv2.1 channel redox state

**Figure 5.**
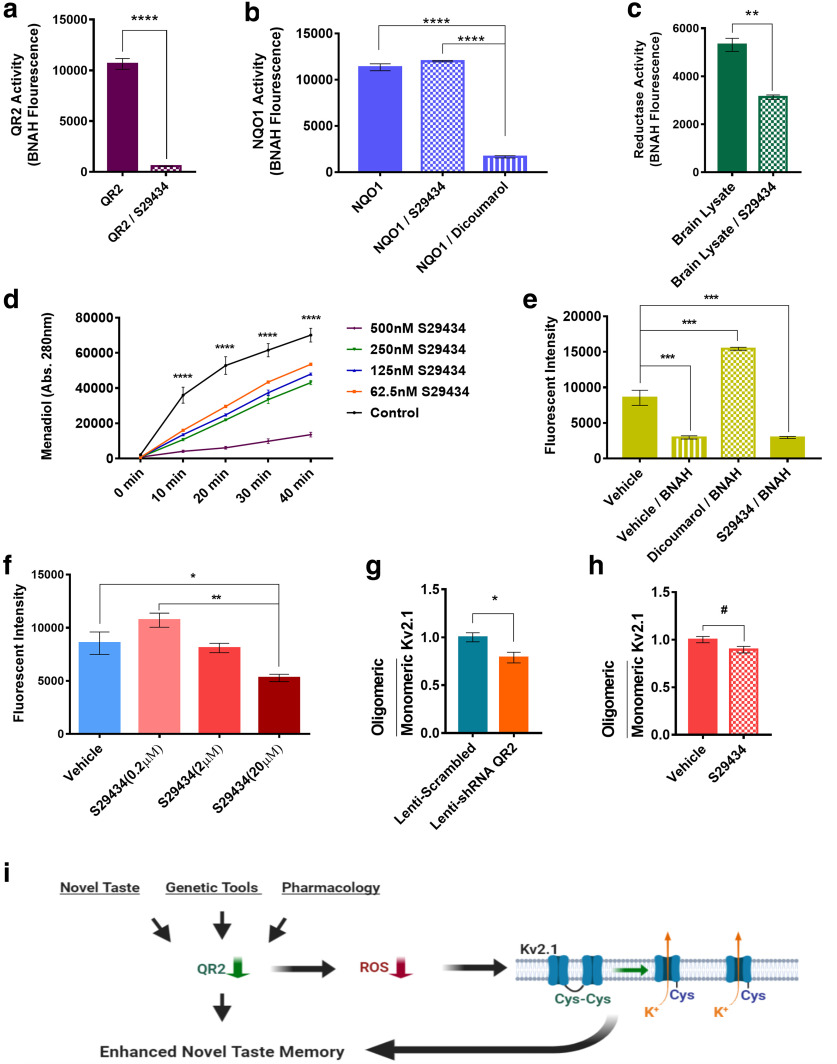
Endogenous QR2 activity generates cellular ROS and affects Kv2.1 channel redox state. ***a***, QR2 activity is strongly inhibited by S29434. ***b***, NQO1 activity is not affected by S29434, but is sensitive to dicoumarol. ***c***, Endogenous mouse brain NQO1 and QR2 activity can be measured with BNAH and is sensitive to QR2 inhibitor S29434. ***d***, QR2 uses small molecules of the mouse brain cytoplasm as cofactors to reduce menadione to menadiol in a S29434-sensitive manner. ***e***, HEK293FT cells given reductase cofactor BNAH show reduction in the ROS signal, which is sensitive to dicoumarol, but not to S29434. ***f***, Basal ROS levels in HEK293FT cells are reduced by S29434 in a dose-dependent manner. ***g***, Kv2.1 clumping in the mouse aIC is significantly reduced by infection with shQR2 compared with scrambled control lentivirus. ***h***, Kv2.1 clumping in the mouse aIC trends toward significant reduction by S29434 compared with vehicle. ***i***, Schematic representation of the molecular events following QR2 downregulation. Upon reduced QR2 expression or activity, either naturally or experimentally, there is a reduction in cellular ROS. In the mouse brain, this is exemplified by the redox state of ROS-sensitive Kv2.1 potassium channels. Although Kv2.1 channels are likely not the only molecules affected by QR2 ROS modulation, functional Kv2.1 conductance has been shown to be important for proper memory formation. Data are shown as the mean ± SEM. #*p* = 0.05; *p < 0.05; **p < 0.01; ***p < 0.001; ****p < 0.0001. See supporting data in Extended Data Figure 5-1 and Extended Data Figure 5-2.

The function of QR2 is poorly understood in cells and is unknown in neurons. To establish whether QR2 can catalyze reduction reactions endogenously in the brain, the previously described QR2 inhibitor S29434 was used (Ferry et al., 2010). To validate its efficacy, purified QR2 activity was measured with or without the inhibitor. In accordance with previous studies, significant reduction in QR2 activity was observed using 200 nm S29434 and 10 nm QR2 (Fig. 5*a*). To ensure that the closely related NQO1 is not affected by a similar dose of S29434, purified NQO1 activity was measured with 200 nm S29434, dicoumarol (NQO1 inhibitor; Timson, 2017), or vehicle.

10.1523/ENEURO.0067-20.2020.f5-1 Figure 5-1 Time-course of menadiol formation by QR2 activity with or without S29434, using endogenous brain cytoplasmic small molecules as cofactors. Download Figure 5-1, TIF file.

10.1523/ENEURO.0067-20.2020.f5-2 Figure 5-2 Cysteine redox in Kv2.1 is sensitive to ROS generated by QR2 activity. ***a***, Kv2.1 blot following nonreducing gel electrophoresis of mouse aIC. Top, bottom, Two separate experiments in which either lentivirus containing shRNA targeting QR2 mRNA (top) or a scrambled control (bottom) was injected into the mouse aIC. ***b***, Kv2.1 blot following nonreducing gel electrophoresis of mouse aIC samples. Top, bottom, Two separate experiments in which either QR2 inhibitor S29434 (top) or vehicle (bottom) was injected intraperitoneally. ***c***, Kv2.1 oligomerization due to aIC redox state, as seen in ***a*** (top) is abolished in the blot following the addition of β-mercaptoethanol. ***d***, Kv2.1 oligomerization due to aIC redox state, as seen in ***b*** (top), is abolished in the blot following the addition of β-mercaptoethanol. Download Figure 5-2, TIF file.

S29434 did not affect NQO1 activity, which was significantly reduced by dicoumarol, its known inhibitor (Fig. 5*b*). Both NQO1 and QR2 are expressed in the mouse brain, and both can use BNAH as a synthetic cofactor, enabling concomitant endogenous brain quinone reductase activity measurement. Mouse brain cytoplasm was thus incubated with BNAH (100 μm) and S29434 (200 nm) or vehicle, and total reductase activity was measured. As seen in Figure 5*c*, BNAH was effectively oxidized by endogenous quinone reductases, whereas S29434 administration significantly diminished the catalysis of BNAH oxidation. These findings demonstrate that, in the mouse brain cytoplasm, QR2 is able to act on quinone substrates (either endogenous or exogenous), given a synthetic cofactor, and that this activity is sensitive to S29434.

A long-standing issue with QR2 is the lack of any known endogenous cofactors, except the possible existence of a NADH metabolite, NRH (Friedlos and Knox, 1992). To assess whether there are any endogenous small molecules that could serve as cofactors in the brain, mouse brain cytoplasm was filtered, resulting in a filtrate containing only molecules ≤3 kDa in size. Pure QR2 can be added to this filtrate, along with the synthetic substrate menadione. Upon QR2 activity, menadione is reduced to menadiol, a process that can be detected using HPLC (Boutin et al., 2005; Extended Data Fig. 5-1). We therefore conducted this assay, using increasing doses of S29434. A clear enzymatic reaction curve was seen, which was effectively inhibited by S29434 in a dose-dependent manner (S29434 doses: 200 nm, 2 μm, and 20 μm; Fig. 5*d*), confirming the presence of endogenous QR2 cofactors in the mouse brain for the first time. Together, these data indicate that QR2 does act as a quinone reductase in the brain, which raises the question as to what outcome.

Previous reports indicate a potentially deleterious effect of QR2 activity (Cassagnes et al., 2015; Chen et al., 2017). We therefore aimed to test the effect of QR2 activity on cellular ROS. To achieve this, HEK293FT quinone reductase activity was elevated by the addition of BNAH, and the effect of NQO1 and QR2 was measured by the addition of either dicoumarol or S29434. Baseline ROS levels were significantly reduced by quinone reductase activation upon the addition of BNAH; however, this was reversed only by the inhibition of NQO1, but not of QR2 (Fig. 5*e*). This raises the possibility that QR2 activity may have a net opposite effect on the redox outcome in relation to that of NQO1, possibly due to the differential substrate specificity (Zhao et al., 1997). To more closely assess the effect of QR2 activity on cellular ROS, HEK293FT cells were treated with increasing doses of S29434. A dose-dependent decrease in ROS was measured (Fig. 5*f*), indicating that QR2 contributes to cellular ROS, which corroborates reports in other cellular models using QR2 inhibitors. We next tested whether QR2 activity affects the redox state in the brain. To do so, we measured the redox state of ion channels expressed on neuronal soma, which have been shown to be involved in cognitive function in an ACh-dependent manner (Mohapatra and Trimmer, 2006; Yu et al., 2016). A promising ion channel to focus on was the voltage-gated potassium channel Kv2.1, which forms oligomers via cysteine oxidation and disulfide bridge formation between the channels on the cell membrane, thereby affecting the proper function of the channel (Misonou et al., 2005). We therefore aimed to measure Kv2.1 oligomerization upon QR2 expression reduction.

Mice were injected to the aIC with either shQR2 or a scrambled control lentivirus. Kv2.1 oligomerization was then measured by native gels and Western blot (Extended Data Fig. 5-2). A significant reduction in Kv2.1 clumping was observed in mice with genetically reduced QR2 expression, compared with scrambled controls (Fig. 5*g*). To examine whether QR2 inhibition by intraperitoneal injection of S29434 has a similar effect on Kv2.1 oligomerization, mice were injected with the inhibitor or vehicle and were killed 4 h later. Mice receiving S29434 also showed a trend of reduced Kv2.1 oligomerization (Fig. 5*h*), albeit this was predictably less effective than local viral infection. Together, we show here that QR2 acts as a quinone reductase in the mouse brain, using endogenous small molecules as cofactors. The net effect of this QR2 activity contributes to cellular ROS, which are removed upon QR2 suppression in the aIC, leading to the observed QR2-dependent stabilization of novel taste memory (Fig. 5*i*).

## Discussion

The current dogma of biological mechanisms underlying learning and memory proposes an active process where sensory information is converted to electrical activity. This activity enters the CNS and forms an internal representation of the world, which can in turn modify previous knowledge (Fernández and Morris, 2018). If the information is important according to evolutionary and developmental selection, this short-term memory trace can be stabilized to form a long-term memory to guide future behavior. These processes are mediated by a sequence of cellular and molecular events beginning with post-translation modifications, followed by proteostasis and gene expression programs enabling the consolidation and stabilization of a memory (Kandel et al., 2014). However, there is another possibility, where stabilization of memories involves the removal of an active constraint. In fact, there are many instances in biology where this strategy is used (e.g., kinase regulatory subunits, action potential thresholds). Here, we show that QR2 expression functions as such a removable memory constraint, describe its upstream mechanisms, and propose a mode of action.

Specifically, we found that QR2 expression is sensitive to local GABA_A_R antagonism, unless mAChRs are antagonized beforehand, revealing a hierarchical, local relationship of GABA release downregulation → ACh release upregulation → activation of mAChR → QR2 expression downregulation on novel taste stimuli (Fig. 2). Furthermore, we show that QR2 expression reduction is a major component of novel taste memory formation and consolidation. Being downstream of ACh, artificially removing QR2 is sufficient to form a long-term safe taste memory in the absence of ACh in the aIC (Fig. 1). Such localized specificity of QR2 reduction in the aIC by means of this disinhibitory circuit reveals QR2 as a central element of novel memory consolidation acting downstream of the known distal and local cellular activities during taste consumption. QR2 ultimately indicates novelty in this distinct sensory modality at the site of the taste memory per se (i.e., the aIC) and acts during the classic molecular consolidation phase. Furthermore, as identified in the study by Rappaport et al. (2015) and corroborated here, this occurs regardless of the taste itself or the associated palatability or aversion, but rather due to the novelty in and of itself. Thus, QR2 functions as a molecular “off” switch that enables the enhanced memory attained following an encounter of an animal with new incidental (i.e., nonassociative) information perceived as important. Local control of ACh release has been demonstrated in other cortical areas during various activities or stimuli as well (Giorgetti et al., 2000). Here, further evidence supporting local control of ACh by GABA sheds light on downstream outcomes of this form of local control of cholinergic modulation. This may indicate that ACh exerts its effects in two distinct phases during novel taste memory formation. The first phase is an immediate one, which occurs via known and well described mechanisms, such as the modulation of neuronal intrinsic properties, the enhancement of afferent input, and synaptic potentiation (Hasselmo, 2006). The second phase, which we described here, is a lingering one, which is mediated by QR2 reduced expression. This may also explain the functionally prolonged local release of ACh seen in the aIC following novel taste consumption (Shimura et al., 1995; Rodríguez-García and Miranda, 2016).

Unlike many other molecules involved in long-term memory formation, the reduction in QR2 mRNA expression levels occurs independently of changes in QR2 transcription (Fig. 3). Instead, QR2 mRNA is degraded following miR-182 upregulation upon novel taste consumption (Figs. 3, 4). The control of miR-182 and QR2 by the same GABA and ACh pathway, in an inverse and stepwise manner, first affecting miR-182 and subsequently affecting QR2, ties both in with the local disinhibitory GABA_A_R/mAChR circuit involved in novel taste memory (Fig. 3). This is an example of the effect of neuromodulation on gene expression, which defines a cortical state (Záborszky et al., 2018; La Camera et al., 2019). Furthermore, this establishes the importance of miR-182 in novel taste learning in the aIC and identifies QR2 as a new target for this miR. The upstream control of GABA_A_R and mAChR on miR-182 in the aIC sheds further light on the mechanisms involved in its induction during long-term memory formation in the cortex. Intriguingly, previous studies have demonstrated the suppression of miR-182 cluster by protein phosphatase 1 (PP1) as a removable constraint on memory formation (Genoux et al., 2002; Woldemichael et al., 2016). The authors describe the reduced expression of miR-182 and the associated cognitive decline as reversible by overexpression of the miR-182 cluster (Jawaid et al., 2019), which is underexpressed during aberrant PP1 activity in aging and neurodegeneration. It is therefore possible that QR2 is placed further downstream as part of the effect of PP1-mediated miR-182 regulation following novel taste stimulus in the aIC (Adaikkan and Rosenblum, 2012).

Despite their structural similarity, QR2 (NQO2) and NQO1 cofactor and substrate affinity is distinctly different, and the inhibitor sensitivity of these two enzymes is equally dissimilar (Antoine et al., 2012). Since no endogenous QR2 cofactor has been confirmed, and previous studies have found varied outcomes of QR2 expression and activity, the function of QR2 remains unclear, especially in the brain, where less research has been done. Furthermore, a few studies have demonstrated the ability of QR2 to produce ROS instead of clearing them, via preferential reduction of orthoquinones, such as the quinone form of dopamine (Fu et al., 2008). Here, we confirm for the first time the presence of QR2 cofactors in the mouse brain, as well as QR2 activity in brain cytoplasm, which is sensitive to S29434 (Fig. 5). We therefore establish that QR2 is an active second quinone reductase in the mouse brain (in addition to NQO1), where there are endogenous small molecules serving as cofactors and substrates on which the enzyme is able to act.

Intriguingly, QR2 activity, as opposed to NQO1 (Dinkova-Kostova and Talalay, 2010), contributes to cellular ROS. There are many ROS generating and clearing enzymes in the brain that under normal physiological conditions have an important role in memory formation (Massaad and Klann, 2011; Sies, 2017). The identification of QR2 as a removable mild ROS contributor and constraint on long-term memory consolidation raises the possibility that QR2 acts via redox modulation following taste novelty. This theory is supported by the decreased clumping of the Kv2.1 voltage-gated potassium channel seen in the mouse brain on QR2 removal (Fig. 5). When clumped, the conductance of Kv2.1 is altered, thus changing the intrinsic properties of neurons (Frazzini et al., 2016). This potentially deleterious phenomenon, which is correlated with memory decline, has been seen to increase in aged animals and in AD model mice (Patel and Sesti, 2016). In light of these data, it may be that QR2 is used to modulate redox to better facilitate transient high-plasticity states in neurons tagged for a memory trace. The oxidation of Kv2.1 represents only one important example of redox modulation in neurons that relates directly to memory (Bogeski and Niemeyer, 2014). However, this does not exclude the possibility that QR2 may affect many other redox-sensitive molecules, as it acts freely in the cytoplasm across the brain. Intriguingly, other well studied molecules that also reduce ROS and improve memory coincidentally inhibit QR2. Both melatonin (Boutin, 2016) and resveratrol (Buryanovskyy et al., 2004) are such molecules. However, it is yet unknown whether QR2 acts similarly in other brain regions in a manner that could explain in part the memory-improving and ROS-clearing effects of these molecules, as they have many other targets (Emet et al., 2016; Berman et al., 2017). Ultimately, it is necessary for further studies to establish the effect of QR2 in other brain regions during different learning modalities, and not just the aIC following taste learning in male subjects.

QR2 is overexpressed in AD brains Hashimoto and Nakai (2011). A major avenue toward tackling memory loss in AD is a multimodal approach involving the targeting of ACh depletion and receptor activation by using ACh esterase inhibitors, ACh precursors, and ACh receptor agonists (Pepeu and Giovannini, 2010; Melancon et al., 2013). Another major avenue toward treating any neurodegenerative disease is reducing inflammation. A parsimonious route to reduce inflammation has been via the enhancement of the cholinergic system (Tabet, 2006). ACh esterase inhibitors and mAChR activation ameliorate inflammation in AD brains, while antagonism of the mAChR system is proinflammatory and maintains high QR2 levels (Fig. 1; Liu et al., 2017). It is therefore an intriguing possibility that QR2 may be at the crux of ACh downregulation and increased inflammation, acting as a potentially harmful reductase in cholinergically depleted cortical systems. Moreover, in our hands, the known cognitive enhancement of ACh esterase inhibitors can be explained by QR2-reduced expression in the cortex (Extended Data Fig. 4-1). Thus, our findings point to QR2 as a promising target for memory enhancement in health and disease.

## Synthesis

Reviewing Editor: Thomas McHugh, RIKEN Center for Brain Science Decisions are customarily a result of the Reviewing Editor and the peer reviewers coming together and discussing their recommendations until a consensus is reached. When revisions are invited, a fact-based synthesis statement explaining their decision and outlining what is needed to prepare a revision will be listed below. The following reviewer(s) agreed to reveal their identity: NONE. Note: If this manuscript was transferred from JNeurosci and a decision was made to accept the manuscript without peer review, a brief statement to this effect will instead be what is listed below.

The authors have fully addressed the comments and concerns expressed by the reviewers at the Journal of Neuroscience.
