# Supplementary material for: Muscarinic-Dependent miR-182 and QR2 Expression Regulation in the Anterior Insula Enables Novel Taste Learning
Source: eNeuro. 2020 May 29;7(3):ENEURO.0067-20.2020. doi: 10.1523/ENEURO.0067-20.2020 (PMC7266141; doi:10.1523/ENEURO.0067-20.2020)
Supplement: Table 1-1 — Detailed Statistical Analysis. Download Table 1-1, DOCX file. [file enu-eN-NWR-0067-20-s10.docx]

**
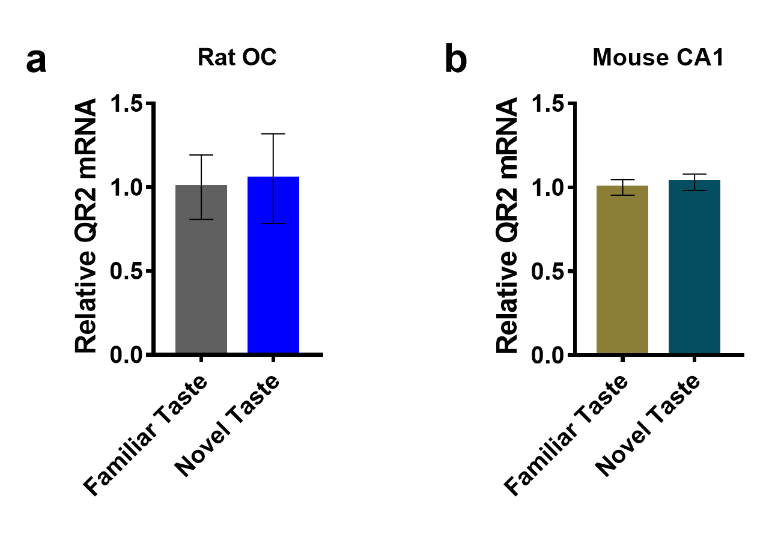
**

**Figure 1-1. QR2 mRNA Expression is Unchanged in Brain Areas Not Associated with Taste Memory Following Novel Taste Consumption.**

**a**) QR2 mRNA is unchanged in rat OC following novel taste consumption. **b**) QR2 mRNA is unchanged in mouse CA1 following novel taste consumption.

**
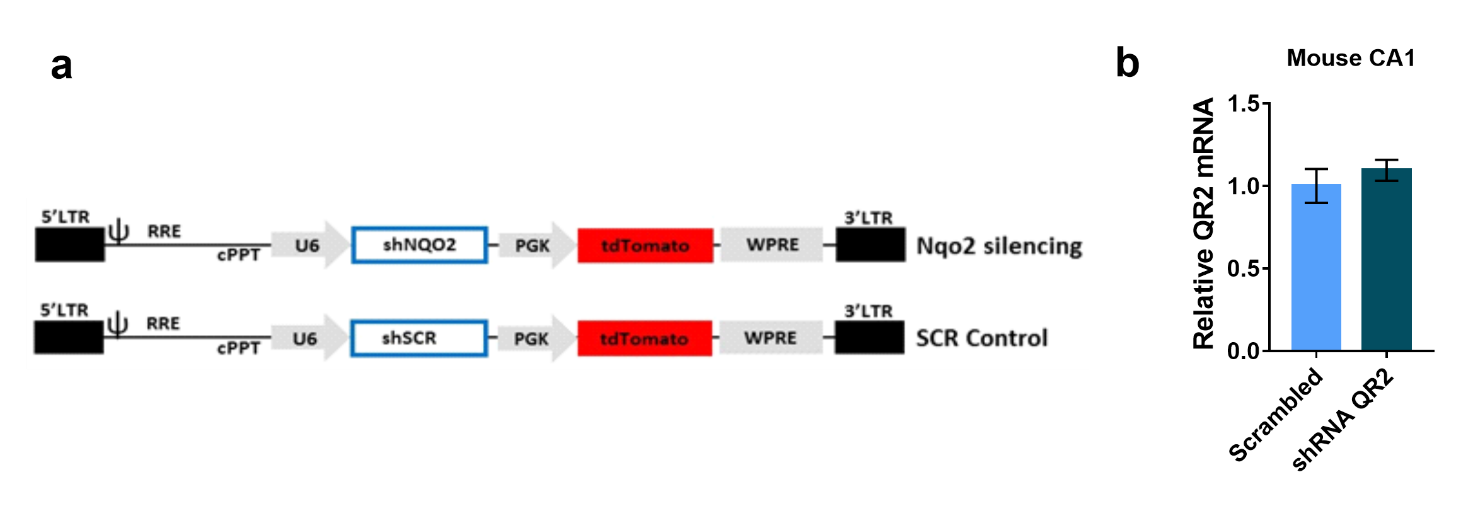
**

**Figure 1-2. Lentivirus Containing Either shRNA Targeting QR2 or a Scrambled Control Injected to aIC Did Not Alter QR2 mRNA Expression in CA1.**

**a**) Diagram of lentivirus containing shRNA targeting QR2, or a scrambled control, used to reduce QR2 expression in mice. **b**) QR2 expression in CA1 remains unaffected by local aIC infection with lentivirus harboring shRNA targeting QR2.

**
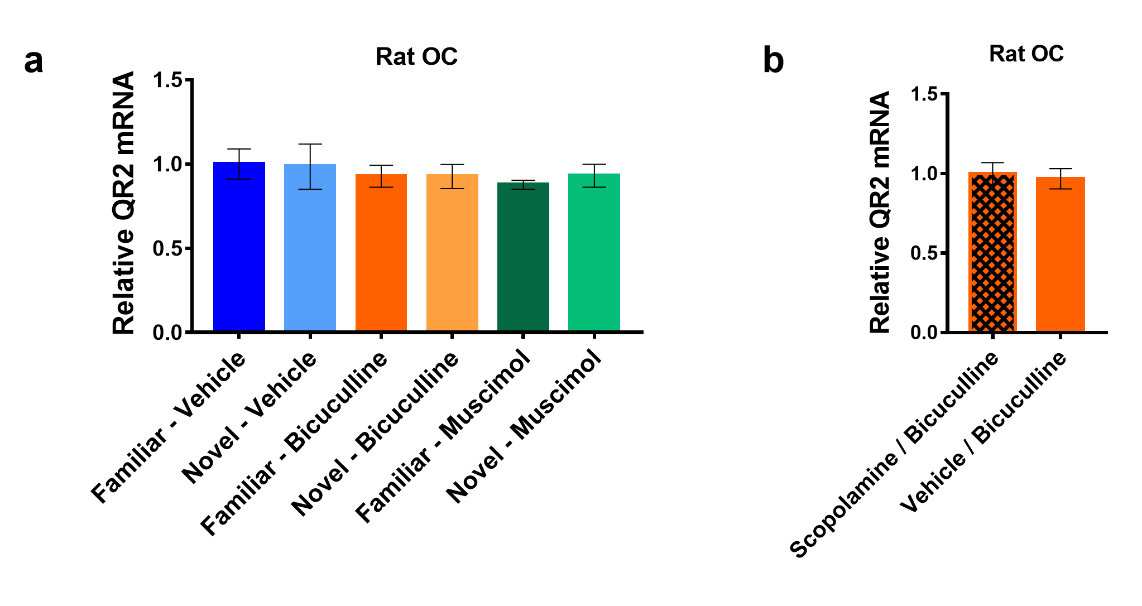
**

**Figure 2-1. Local aIC GABA_A_ Receptor Antagonism and Scopolamine Injections Do Not Affect QR2 mRNA in the Occipital Cortex.**

**a**) QR2 mRNA expression is unchanged following novel taste or antagonism/agonism of GABA_A_R with bicuculline or muscimol locally in the aIC. **b**) QR2 expression remains unchanged in the OC of rats, following local antagonism of GABA_A_R in the aIC, with or without prior injections of scopolamine.

**
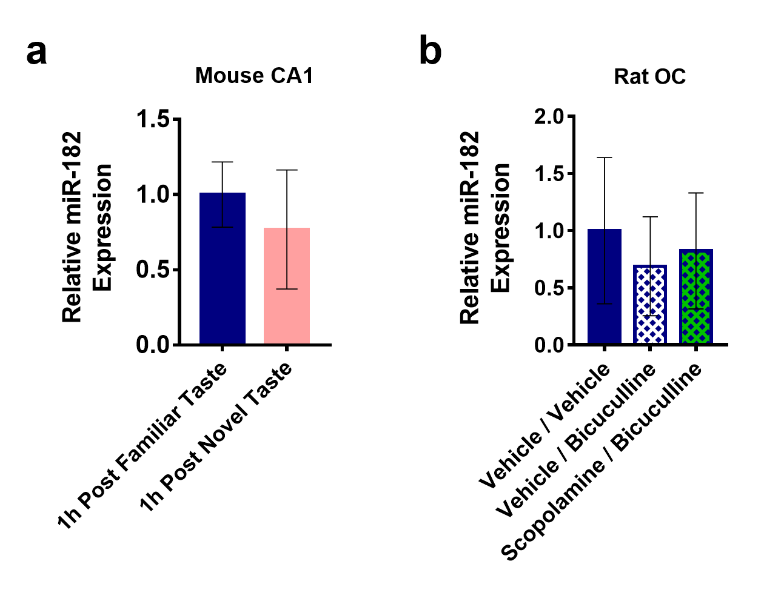
**

**Figure 3-1. miR-182 Expression Does Not Increase in the Mouse CA1 Following Novel Taste Consumption or in the Rat OC Following Pharmacological Manipulation Locally to the Rat aIC.**

**a**) miR-182 levels remain unaltered in the CA1 of mice following novel taste consumption. **b**) miR-182 levels remain unchanged in the OC of rats, following local aIC antagonism of GABA_A_R with bicuculline, with or without prior injection of scopolamine.

**Figure 3-2. Predicted Hybridization Sites of miR-182 to QR2 mRNA in the Human, Mouse, and Rat Genome.
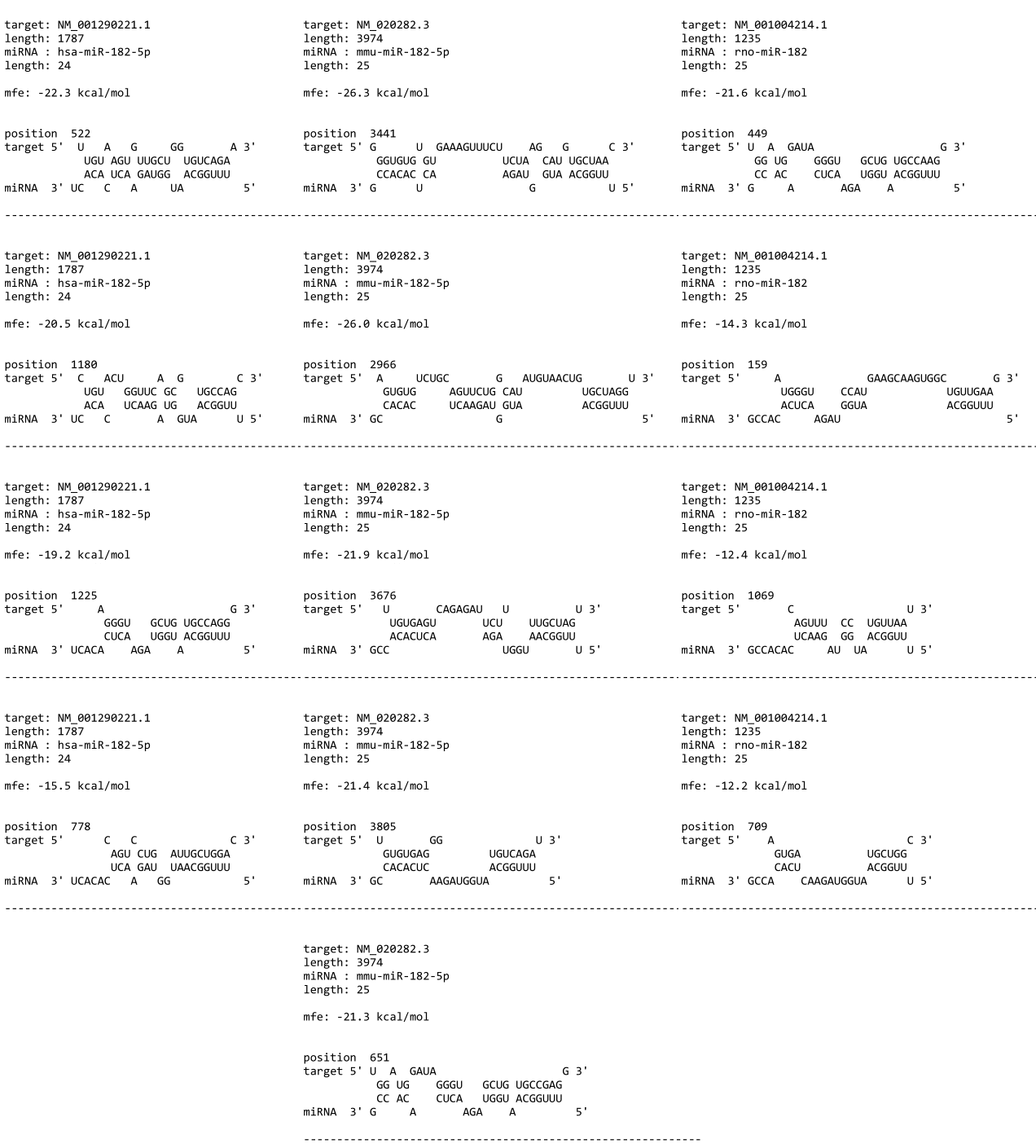
**

**
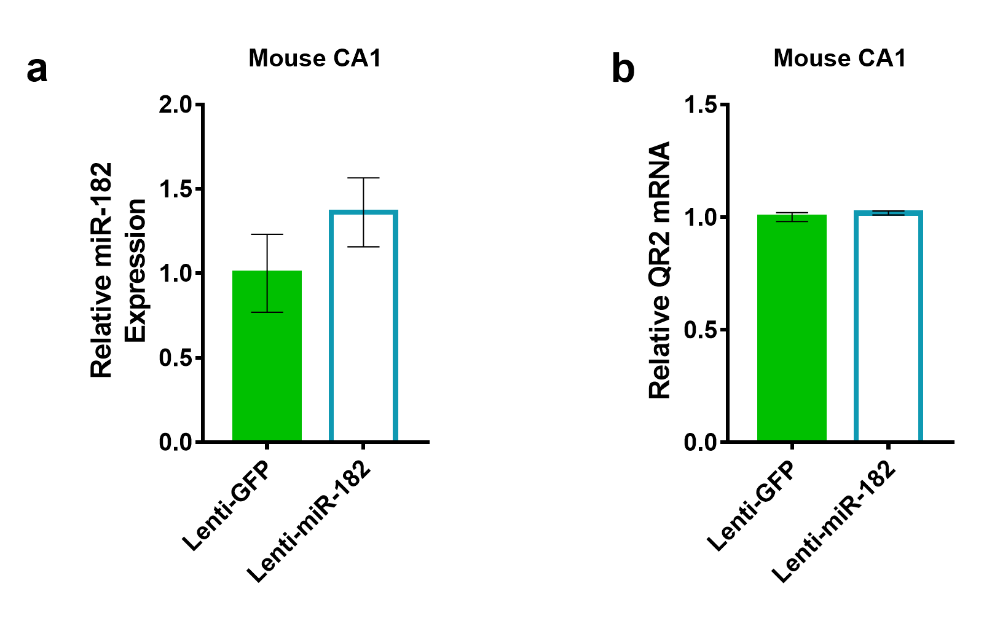
**

**Figure 4-1. Mice Injected to IC with a Lentivirus Expressing miR-182 Do Not Show Changes in QR2 mRNA or miR-182 Expression in the Hippocampus CA1.**

**a**) miR-182 levels in CA1 were not elevated following local aIC infection with a lentivirus overexpressing miR-182. **b**) QR2 mRNA levels in CA1 were not changed following local aIC infection with a lentivirus overexpressing miR-182.

**
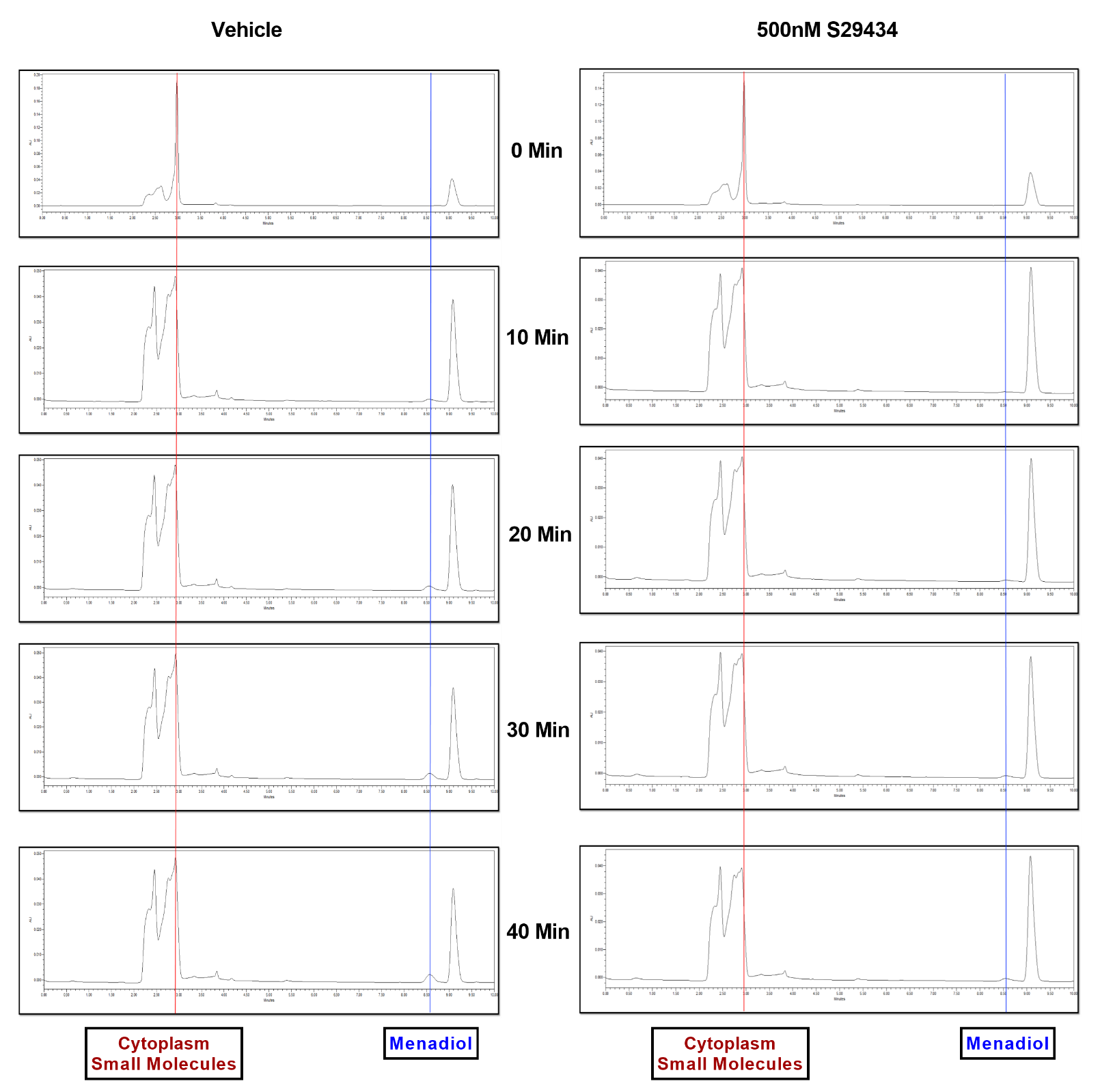
** **Figure 5-1. Time-Course of Menadiol Formation by QR2 Activity with or without S29434, Using Endogenous Brain Cytoplasmic Small Molecules as Co-Factors.**

**
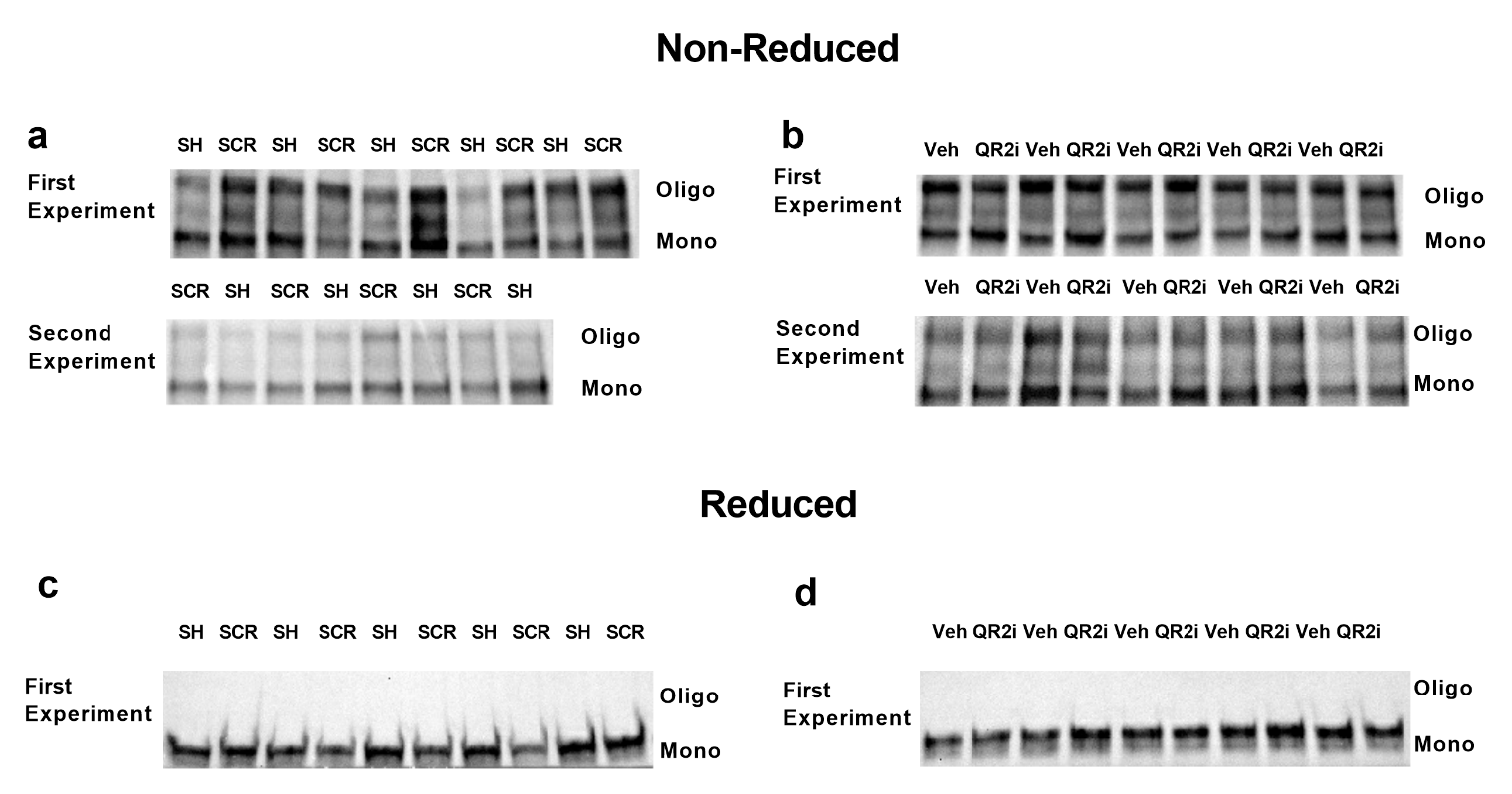
**

**Figure 5-2. Cysteine Redox in Kv2.1 is Sensitive to ROS Generated by QR2 Activity.**

**a**) Kv2.1 blot following non reducing gel electrophoresis of mouse aIC. Top panel and bottom panel represent two separate experiments in which either lentivirus containing shRNA targeting QR2 mRNA or a scrambled control were injected to the mouse aIC. **b**) Kv2.1 blot following non reducing gel electrophoresis of mouse aIC samples. Top panel and bottom panel represent two separate experiments in which either QR2 inhibitor S29434 or vehicle were injected i.p. **c**) Kv2.1 oligomerization due to aIC redox state, as seen in **a**) upper panel, is abolished in the blot following the addition of β mercaptoethanol. **d**) Kv2.1 oligomerization due to aIC redox state, as seen in **b**) upper panel, is abolished in the blot following the addition of β mercaptoethanol.

**Table 1-1. Detailed Statistical Analysis.**

| **Figure** | **Statistical analysis** | **Post hoc tests** |
| --- | --- | --- |
| **Fig. 1b** | Familiar 3.21 ± 0.113 ΔCt, n=5; Novel 3.996 ± 0.135 ΔCt, n=4; **Student's t test**, t=4.497, df=7, p=0.0028 |  |
| **Fig. 1c** | Familiar 4.975 ± 0.041 ΔCt, n=7; Novel 5.261 ± 0.095 ΔCt, n=7; **Student's t test**, t=2.751 df=12, p=0.0176 |  |
| **Fig. 1e** | Scrambled 39.22 ± 7.538 %, n=4; shRNA to QR2 84.57 ± 2.381 %, n=5, **Student’s t test**, t=6.342 df=7, p=0.0004 |  |
| **Fig. 1 f** | Scrambled 4.513 ± 0.048 ΔCt, n=4; shRNA QR2 4.784 ± 0.063 ΔCt, n=5; **Student's t test**, t=3.263, df=7, p=0.0138 |  |
| **Fig. 1i** | Vehicle 59.96 ± 7.296%, n=7; S29434 83.45 ± 4.109%, n=7; S29434 Post Acq. 78.45 ± 2.813%, n=7; **one way ANOVA**, F 1 (2, 18) = 5.886, p=0.0108 | **Tukey’s multiple comparison test**, Vehicle vs. S29434 p=0.0116, Vehicle vs. S29434 Post Acq. P=0.0489 |
| **Fig. 1k** | Vehicle/Vehicle 62.67 ± 5.749%, n=11; Scopolamine/Vehicle 37.35 ± 6.476%, n=10; Scopolamine/S29434 65.49 ± 6.029%, n=10; **Kruskal-Wallis**, p=0.0139 | **Dunn’s multiple comparison**, Vehicle/Vehicle vs. Scopolamine/Vehicle p=0.0406, Vehicle/Vehicle vs. Scopolamine/S29434 p>0.999, Scopolamine/Vehicle vs Scopolamine/S29434 p=0.0274 |

| **Fig. 2b** | Vehicle – Novel 6.005 ± 0.259 ΔCt, n=6; Vehicle – Familiar 5.558 ± 0.137 ΔCt, n=4; Bicuculline – Novel 6.082 ± 0.057 ΔCt, n=5; Bicuculline – Familiar 6.125 ± 0.267 ΔCt, n=6; Muscimol – Novel 5.848 ± 0.289 ΔCt, n=5; Muscimol – Familiar 5.837 ± 0.163 ΔCt, n=5; **Two way ANOVA**, Treatment F=6.343 p=0.006, Taste F=3.026 p=0.094, Interaction F=3.718 p=0.039. | **Tukey’s multiple comparison test for treatment**, Vehicle vs. Bicuculline p=0.02, Vehicle vs. Muscimol p=0.985, Bicuculline vs Muscimol p=0.029.  **ANOVA test of between subject effects**, Vehicle – Familiar vs. Vehicle – Novel p=0.014, Bicuculline – Familiar vs. Bicuculline – Novel p=0.735, Muscimol – Familiar vs. Muscimol - Novel p=0.942. |
| --- | --- | --- |
| **Fig. 2e** | Scopolamine/Bicuculline 7.372 ± 0.056 ΔCt, n=8; Vehicle/Bicuculline 7.746 ± 0.119 ΔCt, n=6; **Student's t test**, t=3.087 df=12, p=0.0094 |  |
| **Fig. 2g** | Vehicle 73.28 ± 4.962%, n=8; Bicuculline 87.95 ± 2.849%, n=8; **Student's t test**, t=2.564 df=14, p=0.0225 |  |
| **Fig. 3a** | Familiar 7.377 ± 0.067 ΔCt, n=6; Novel 7.605 ± 0.122 ΔCt, n=6; **Student's t test**, t=1.637 df=10, p=0.1326 |  |
| **Fig. 3b** | Familiar 1 h 12.49 ± 0.248 ΔCt, n=8; Novel 1 h 11.2 ± 0.355 ΔCt, n=8; **Mann-Whitney test**, p=0.007  Familiar 3 h 13.08 ± 0.446 ΔCt, n=7; Novel 3 h 12.68 ± 0.548 ΔCt, n=7; **Student's t test**, t=0.5573 df=12, p=0.5876 |  |
| **Fig. 3d** | Vehicle/Vehicle 17.06 ± 0.224 ΔCt, n=6; Vehicle/Bicuculline 15.86 ± 0.254 ΔCt, n=6; Scopolamine/Bicuculline 16.63 ± 0.296 ΔCt, n=6; **Kruskal-Wallis test**, p=0.0147 | **Dunn’s multiple comparison test**, Vehicle/Vehicle vs. Vehicle/Bicuculline p=0.0206, Vehicle/Vehicle vs. Scopolamine/Bicuculline p>0.999, Vehicle/Bicuculline vs. Scopolamine/Bicuculline p=0.198 |
| **Fig. 3f** | Vehicle/Vehicle 17.45 ± 0.217 ΔCt, n=5; Vehicle/Eserine 15.99 ± 0.2662 ΔCt, n=5; **Student's t test**, t=4.241 df=8, p=0.0028 |  |
| **Fig. 4b** | GFP 10.79 ± 0.162 ΔCt, n=6; miR-182 5.396 ± 1.522 ΔCt, n=4; **Mann-Whitney test**, p=0.0095 |  |
| **Fig. 4c** | GFP 6.576 ± 0.02059 ΔCt, n=6; miR-182 6.864 ± 0.03825 ΔCt, n=4; **Student's t test**, t=7.239 df=8, p<0.0001 |  |
| **Fig. 4e** | Lenti-GFP 27.17 ± 2.913 %, n=5; Lenti-miR-182 60.63 ± 6.026 %, n=5; **Student's t test**, t=5 df=8, p=0.0011 |  |
| **Fig. 4f** | Lenti-GFP 10.92 ± 0.436 ΔCt, n=5; Lenti-miR-182 8.441 ± 0.341 ΔCt, n=4; **Student's t test**, t=4.277 df=7, p=0.0037 |  |
| **Fig. 4g** | Lenti-GFP 9.145 ± 0.068 ΔCt, n=5; Lenti-miR-182 9.4 ± 0.075 ΔCt, n=4; **Student's t test**, t=2.509 df=7, p=0.0405 |  |
| **Fig. 5a** | QR2 10,643 ± 529.4 AU, n=3; QR2/S29434 569.7 ± 13.97 AU, n=3; **Student's t test**, t=19.02 df=4, p<0.0001 |  |
| **Fig. 5b** | NQO1 11,343 ± 376.7 AU, n=3; NQO1/S29434 11,997 ± 71.39 AU, n=3; NQO1/Dicoumarol 1,409 ± 124.9 AU, n=3; **one way ANOVA**, F (2, 6) = 619.4, p<0.0001 | **Tukey’s multiple comparison test**, NQO1 vs. NQO1/S29434 p=0.1958, NQO1 vs. NQO1/Dicoumarol p<0.0001, NQO1/S29434 vs. NQO1/Dicoumarol p<0.0001 |
| **Fig. 5c** | Brain Lysate 4,834 ± 271.6 AU, n=3; Brain Lysate/S29434 2,967 ± 91.83 AU, n=3; **Student's t test**, t=7.616 df=4, p=0.0016 |  |
| **Fig. 5d** | **Two-way repeated measures ANOVA**, Dose F (4, 11) = 91.3, p<0.0001; Interaction F (16, 44) = 56.49, p<0.0001; Time F (4, 44) = 1074, p<0.0001; Subjects F (11, 44) = 7.616, p<0.0001; | **Tukey's multiple comparison** control vs. all treated groups p<0.0001 during 10 – 40 min (complete data in Figure 5d Post Hoc Analysis) |
| **Fig. 5e** | Vehicle 8,539 ± 1,057 AU, n=3; Vehicle/BNAH 2,966 ± 218.5 AU, n=3; Dicoumarol/BNAH 15,024 ± 208.2 AU, n=3; S29434/BNAH 2,768 ± 154.4 AU, n=3; **one-way ANOVA**, p<0.0001 | **Tukey’s multiple comparisons test** Vehicle vs. Vehicle/BNAH p=0.0005, Vehicle vs. Dicoumarol/BNAH p=0.0001, Vehicle vs. S29434/BNAH p=0.0005, Vehicle/BNAH vs. Dicoumarol/BNAH p<0.0001, Vehicle/BNAH vs. S29434/BNAH p>0.9999, Dicoumarol/BNAH vs. S29434/BNAH p<0.0001 |
| **Fig. 5f** | Vehicle 8,539 ± 1,057 AU, n=3; S29434 (0.2µM) 10,709 ± 662.8 AU, n=3; S29434 (2µM) 8,090 ± 440.1 AU, n=3; S29434 (20µM) 4,644 ± 344.2 AU, n=3; **one way ANOVA**, p=0.0036 | **Tukey’s multiple comparisons test** Vehicle vs. S29434 (0.2µM) p=0.1909, Vehicle vs. S29434 (2µM) p=0.9647, Vehicle vs. S29434 (20µM) p=0.0390, S29434 (0.2µM) vs. S29434 (2µM) p=0.1002, S29434 (0.2µM) vs. S29434 (20µM) p=0.0022, S29434(2µM) vs. S29434 (20µM) p=0.0750 |
| **Fig. 5g** | Lenti-Scrambled 1 ± 0.04766 AU, n=9; Lenti-shRNA QR2 0.7881 ± 0.05523 AU, n=9; **Mann-Whitney test**, p=0.0106 |  |
| **Fig. 5h** | Vehicle 1 ± 0.03299 AU, n=10; S29434 0.8947 ± 0.03452 AU, n=10; **Mann-Whitney test**, p= 0.0524 |  |
| **Extended Data Fig. 1a** | Familiar 1.757 ± 0.2225 ΔCt, n=4; Novel 1.685 ± 0.308 ΔCt, n=4**; Student's t test**, t=0.1886 df=6, p=0.8566 |  |
| **Extended Data Fig. 1b** | Familiar 4.286 ± 0.05 ΔCt, n=7; Novel 4.242 ± 0.053 ΔCt, n=7; **Student's t test**, t=0.6052 df=12, p=0.5563 |  |
| **Extended Data Fig. 2b** | Scrambled 5.024 ± 0.118 ΔCt, n=4; shRNA QR2 4.893 ± 0.072 ΔCt, n=5; **Student's t test**, t=0.9903 df=7, p=0.355 |  |
| **Extended Data Fig. 3a** | Familiar-Vehicle 8.103 ± 0.1035 ΔCt, n=4; Novel-Vehicle 8.126 ± 0.147 ΔCt, n=6; Familiar-Bicuculline 8.212 ± 0.07281 ΔCt, n=5; Novel-Bicuculline 8.214 ± 0.07981 ΔCt, n=5; Familiar-Muscimol 8.293 ± 0.03054 ΔCt, n=4; Novel-Muscimol 8.207 ± 0.07616 ΔCt, n=5; **Two way ANOVA,** Taste F=0.060 p=0.809, Treatment F=0.934 p=0.407, Interaction F=0.155 p=0.857 |  |
| **Extended Data Fig. 3b** | Scopolamine/Bicuculline 8.118 ± 0.0731 ΔCt, n=8; Veh/Bic 8.166 ± 0.07022 ΔCt, n=6; **Mann-Whitney test**, p=0.8518 |  |
| **Extended Data Fig. 4a** | 1 h Post Familiar Taste Hippocampus 16.65 ± 0.2314 ΔCt, n=8; 1 h Post Novel Taste Hippocampus 17.03 ± 0.422 ΔCt, n=8; **Student t test**, t=0.7885 df=14, p=0.4435 |  |
| **Extended Data Fig. 4b** | Vehicle / Vehicle 13.14 ± 0.715 ΔCt, n=5; Vehicle / Bicuculline 13.68 ± 0.474 ΔCt, n=6; Scopolamine / Bicuculline 13.42 ± 0.554 ΔCt, n=6; **Kruskal-Wallis test**, p=0.8791 | **Dunn’s multiple comparison test**, Vehicle / Vehicle vs. Vehicle / Bicuculline p>0.9999, Vehicle / Vehicle vs. Scopolamine / Bicuculline p>0.9999, Vehicle / Bicuculline vs. Scopolamine / Bicuculline p>0.9999 |
| **Extended Data Fig. 5a** | Lenti-GFP Hippocampus 15.72 ± 0.2585 ΔCt, n=5; Lenti-miR-182 Hippocampus 15.27 ± 0.235 ΔCt, n=4; **Student t test**, t=1.239 df=7, p=0.2554 |  |
| **Extended Data Fig. 5b** | Lenti-GFP Hippocampus 8.814 ± 0.022 ΔCt, n=5; Lenti-miR-182 Hippocampus 8.788 ± 0.010 ΔCt, n=4; **Student t test**, t=0.986 df=7, p=0.3568 |  |

**Figure 5d Post Hoc Analysis.**

| *Tukey's multiple comparisons test* | *Mean Diff.* | *95.00% CI of diff.* | *Significant?* | *Summary* | *Adjusted P Value* |  |  |  |
| --- | --- | --- | --- | --- | --- | --- | --- | --- |
|  |  |  |  |  |  |  |  |  |
| *0 min* |  |  |  |  |  |  |  |  |
| *Control vs. 62.5nM S29434* | *1446* | *-6013 to 8905* | *No* | *ns* | *0.9819* |  |  |  |
| *Control vs. 125nM S29434* | *1374* | *-6084 to 8833* | *No* | *ns* | *0.9850* |  |  |  |
| *Control vs. 250nM S29434* | *1362* | *-6097 to 8821* | *No* | *ns* | *0.9855* |  |  |  |
| *Control vs. 500nM S29434* | *1318* | *-5659 to 8295* | *No* | *ns* | *0.9836* |  |  |  |
| *62.5nM S29434 vs. 125nM S29434* | *-71.67* | *-7530 to 7387* | *No* | *ns* | *>0.9999* |  |  |  |
| *62.5nM S29434 vs. 250nM S29434* | *-84* | *-7543 to 7375* | *No* | *ns* | *>0.9999* |  |  |  |
| *62.5nM S29434 vs. 500nM S29434* | *-128.3* | *-7105 to 6849* | *No* | *ns* | *>0.9999* |  |  |  |
| *125nM S29434 vs. 250nM S29434* | *-12.33* | *-7471 to 7446* | *No* | *ns* | *>0.9999* |  |  |  |
| *125nM S29434 vs. 500nM S29434* | *-56.67* | *-7034 to 6920* | *No* | *ns* | *>0.9999* |  |  |  |
| *250nM S29434 vs. 500nM S29434* | *-44.33* | *-7021 to 6933* | *No* | *ns* | *>0.9999* |  |  |  |
|  |  |  |  |  |  |  |  |  |
| *10 min* |  |  |  |  |  |  |  |  |
| *Control vs. 62.5nM S29434* | *19949* | *12490 to 27408* | *Yes* | ****** | *<0.0001* |  |  |  |
| *Control vs. 125nM S29434* | *22421* | *14962 to 29880* | *Yes* | ****** | *<0.0001* |  |  |  |
| *Control vs. 250nM S29434* | *25220* | *17761 to 32679* | *Yes* | ****** | *<0.0001* |  |  |  |
| *Control vs. 500nM S29434* | *31852* | *24875 to 38829* | *Yes* | ****** | *<0.0001* |  |  |  |
| *62.5nM S29434 vs. 125nM S29434* | *2472* | *-4987 to 9931* | *No* | *ns* | *0.8821* |  |  |  |
| *62.5nM S29434 vs. 250nM S29434* | *5271* | *-2188 to 12730* | *No* | *ns* | *0.2829* |  |  |  |
| *62.5nM S29434 vs. 500nM S29434* | *11903* | *4926 to 18880* | *Yes* | ***** | *0.0001* |  |  |  |
| *125nM S29434 vs. 250nM S29434* | *2799* | *-4660 to 10258* | *No* | *ns* | *0.8267* |  |  |  |
| *125nM S29434 vs. 500nM S29434* | *9431* | *2454 to 16408* | *Yes* | **** | *0.0031* |  |  |  |
| *250nM S29434 vs. 500nM S29434* | *6632* | *-345 to 13609* | *No* | *ns* | *0.0699* |  |  |  |
|  |  |  |  |  |  |  |  |  |
| *20 min* |  |  |  |  |  |  |  |  |
| *Control vs. 62.5nM S29434* | *23239* | *15781 to 30698* | *Yes* | ****** | *<0.0001* |  |  |  |
| *Control vs. 125nM S29434* | *28041* | *20582 to 35500* | *Yes* | ****** | *<0.0001* |  |  |  |
| *Control vs. 250nM S29434* | *30954* | *23496 to 38413* | *Yes* | ****** | *<0.0001* |  |  |  |
| *Control vs. 500nM S29434* | *46702* | *39725 to 53679* | *Yes* | ****** | *<0.0001* |  |  |  |
| *62.5nM S29434 vs. 125nM S29434* | *4802* | *-2657 to 12260* | *No* | *ns* | *0.3752* |  |  |  |
| *62.5nM S29434 vs. 250nM S29434* | *7715* | *256.4 to 15174* | *Yes* | *** | *0.0392* |  |  |  |
| *62.5nM S29434 vs. 500nM S29434* | *23463* | *16486 to 30440* | *Yes* | ****** | *<0.0001* |  |  |  |
| *125nM S29434 vs. 250nM S29434* | *2913* | *-4545 to 10372* | *No* | *ns* | *0.8049* |  |  |  |
| *125nM S29434 vs. 500nM S29434* | *18661* | *11684 to 25638* | *Yes* | ****** | *<0.0001* |  |  |  |
| *250nM S29434 vs. 500nM S29434* | *15748* | *8771 to 22725* | *Yes* | ****** | *<0.0001* |  |  |  |
|  |  |  |  |  |  |  |  |  |
| *30 min* |  |  |  |  |  |  |  |  |
| *Control vs. 62.5nM S29434* | *18079* | *10621 to 25538* | *Yes* | ****** | *<0.0001* |  |  |  |
| *Control vs. 125nM S29434* | *24152* | *16694 to 31611* | *Yes* | ****** | *<0.0001* |  |  |  |
| *Control vs. 250nM S29434* | *28094* | *20635 to 35552* | *Yes* | ****** | *<0.0001* |  |  |  |
| *Control vs. 500nM S29434* | *51729* | *44753 to 58706* | *Yes* | ****** | *<0.0001* |  |  |  |
| *62.5nM S29434 vs. 125nM S29434* | *6073* | *-1386 to 13532* | *No* | *ns* | *0.1616* |  |  |  |
| *62.5nM S29434 vs. 250nM S29434* | *10014* | *2556 to 17473* | *Yes* | **** | *0.0034* |  |  |  |
| *62.5nM S29434 vs. 500nM S29434* | *33650* | *26673 to 40627* | *Yes* | ****** | *<0.0001* |  |  |  |
| *125nM S29434 vs. 250nM S29434* | *3941* | *-3517 to 11400* | *No* | *ns* | *0.5730* |  |  |  |
| *125nM S29434 vs. 500nM S29434* | *27577* | *20600 to 34554* | *Yes* | ****** | *<0.0001* |  |  |  |
| *250nM S29434 vs. 500nM S29434* | *23636* | *16659 to 30613* | *Yes* | ****** | *<0.0001* |  |  |  |
|  |  |  |  |  |  |  |  |  |
| *40 min* |  |  |  |  |  |  |  |  |
| *Control vs. 62.5nM S29434* | *16579* | *9120 to 24037* | *Yes* | ****** | *<0.0001* |  |  |  |
| *Control vs. 125nM S29434* | *22147* | *14689 to 29606* | *Yes* | ****** | *<0.0001* |  |  |  |
| *Control vs. 250nM S29434* | *26967* | *19508 to 34426* | *Yes* | ****** | *<0.0001* |  |  |  |
| *Control vs. 500nM S29434* | *56510* | *49533 to 63487* | *Yes* | ****** | *<0.0001* |  |  |  |
| *62.5nM S29434 vs. 125nM S29434* | *5569* | *-1890 to 13027* | *No* | *ns* | *0.2324* |  |  |  |
| *62.5nM S29434 vs. 250nM S29434* | *10388* | *2930 to 17847* | *Yes* | **** | *0.0022* |  |  |  |
| *62.5nM S29434 vs. 500nM S29434* | *39932* | *32955 to 46908* | *Yes* | ****** | *<0.0001* |  |  |  |
| *125nM S29434 vs. 250nM S29434* | *4820* | *-2639 to 12278* | *No* | *ns* | *0.3714* |  |  |  |
| *125nM S29434 vs. 500nM S29434* | *34363* | *27386 to 41340* | *Yes* | ****** | *<0.0001* |  |  |  |
| *250nM S29434 vs. 500nM S29434* | *29543* | *22566 to 36520* | *Yes* | ****** | *<0.0001* |  |  |  |
|  |  |  |  |  |  |  |  |  |
|  |  |  |  |  |  |  |  |  |
| *Test details* | *Mean 1* | *Mean 2* | *Mean Diff.* | *SE of diff.* | *N1* | *N2* | *q* | *DF* |
|  |  |  |  |  |  |  |  |  |
| *0 min* |  |  |  |  |  |  |  |  |
| *Control vs. 62.5nM S29434* | *2179* | *732.7* | *1446* | *2645* | *3* | *3* | *0.7733* | *55* |
| *Control vs. 125nM S29434* | *2179* | *804.3* | *1374* | *2645* | *3* | *3* | *0.7349* | *55* |
| *Control vs. 250nM S29434* | *2179* | *816.7* | *1362* | *2645* | *3* | *3* | *0.7283* | *55* |
| *Control vs. 500nM S29434* | *2179* | *861* | *1318* | *2474* | *3* | *4* | *0.7533* | *55* |
| *62.5nM S29434 vs. 125nM S29434* | *732.7* | *804.3* | *-71.67* | *2645* | *3* | *3* | *0.03832* | *55* |
| *62.5nM S29434 vs. 250nM S29434* | *732.7* | *816.7* | *-84* | *2645* | *3* | *3* | *0.04492* | *55* |
| *62.5nM S29434 vs. 500nM S29434* | *732.7* | *861* | *-128.3* | *2474* | *3* | *4* | *0.07337* | *55* |
| *125nM S29434 vs. 250nM S29434* | *804.3* | *816.7* | *-12.33* | *2645* | *3* | *3* | *0.006595* | *55* |
| *125nM S29434 vs. 500nM S29434* | *804.3* | *861* | *-56.67* | *2474* | *3* | *4* | *0.0324* | *55* |
| *250nM S29434 vs. 500nM S29434* | *816.7* | *861* | *-44.33* | *2474* | *3* | *4* | *0.02534* | *55* |
|  |  |  |  |  |  |  |  |  |
| *10 min* |  |  |  |  |  |  |  |  |
| *Control vs. 62.5nM S29434* | *35972* | *16023* | *19949* | *2645* | *3* | *3* | *10.67* | *55* |
| *Control vs. 125nM S29434* | *35972* | *13551* | *22421* | *2645* | *3* | *3* | *11.99* | *55* |
| *Control vs. 250nM S29434* | *35972* | *10752* | *25220* | *2645* | *3* | *3* | *13.49* | *55* |
| *Control vs. 500nM S29434* | *35972* | *4121* | *31852* | *2474* | *3* | *4* | *18.21* | *55* |
| *62.5nM S29434 vs. 125nM S29434* | *16023* | *13551* | *2472* | *2645* | *3* | *3* | *1.322* | *55* |
| *62.5nM S29434 vs. 250nM S29434* | *16023* | *10752* | *5271* | *2645* | *3* | *3* | *2.819* | *55* |
| *62.5nM S29434 vs. 500nM S29434* | *16023* | *4121* | *11903* | *2474* | *3* | *4* | *6.805* | *55* |
| *125nM S29434 vs. 250nM S29434* | *13551* | *10752* | *2799* | *2645* | *3* | *3* | *1.497* | *55* |
| *125nM S29434 vs. 500nM S29434* | *13551* | *4121* | *9431* | *2474* | *3* | *4* | *5.391* | *55* |
| *250nM S29434 vs. 500nM S29434* | *10752* | *4121* | *6632* | *2474* | *3* | *4* | *3.791* | *55* |
|  |  |  |  |  |  |  |  |  |
| *20 min* |  |  |  |  |  |  |  |  |
| *Control vs. 62.5nM S29434* | *52825* | *29586* | *23239* | *2645* | *3* | *3* | *12.43* | *55* |
| *Control vs. 125nM S29434* | *52825* | *24784* | *28041* | *2645* | *3* | *3* | *15* | *55* |
| *Control vs. 250nM S29434* | *52825* | *21871* | *30954* | *2645* | *3* | *3* | *16.55* | *55* |
| *Control vs. 500nM S29434* | *52825* | *6123* | *46702* | *2474* | *3* | *4* | *26.7* | *55* |
| *62.5nM S29434 vs. 125nM S29434* | *29586* | *24784* | *4802* | *2645* | *3* | *3* | *2.568* | *55* |
| *62.5nM S29434 vs. 250nM S29434* | *29586* | *21871* | *7715* | *2645* | *3* | *3* | *4.126* | *55* |
| *62.5nM S29434 vs. 500nM S29434* | *29586* | *6123* | *23463* | *2474* | *3* | *4* | *13.41* | *55* |
| *125nM S29434 vs. 250nM S29434* | *24784* | *21871* | *2913* | *2645* | *3* | *3* | *1.558* | *55* |
| *125nM S29434 vs. 500nM S29434* | *24784* | *6123* | *18661* | *2474* | *3* | *4* | *10.67* | *55* |
| *250nM S29434 vs. 500nM S29434* | *21871* | *6123* | *15748* | *2474* | *3* | *4* | *9.003* | *55* |
|  |  |  |  |  |  |  |  |  |
| *30 min* |  |  |  |  |  |  |  |  |
| *Control vs. 62.5nM S29434* | *61535* | *43455* | *18079* | *2645* | *3* | *3* | *9.668* | *55* |
| *Control vs. 125nM S29434* | *61535* | *37382* | *24152* | *2645* | *3* | *3* | *12.92* | *55* |
| *Control vs. 250nM S29434* | *61535* | *33441* | *28094* | *2645* | *3* | *3* | *15.02* | *55* |
| *Control vs. 500nM S29434* | *61535* | *9805* | *51729* | *2474* | *3* | *4* | *29.57* | *55* |
| *62.5nM S29434 vs. 125nM S29434* | *43455* | *37382* | *6073* | *2645* | *3* | *3* | *3.248* | *55* |
| *62.5nM S29434 vs. 250nM S29434* | *43455* | *33441* | *10014* | *2645* | *3* | *3* | *5.355* | *55* |
| *62.5nM S29434 vs. 500nM S29434* | *43455* | *9805* | *33650* | *2474* | *3* | *4* | *19.24* | *55* |
| *125nM S29434 vs. 250nM S29434* | *37382* | *33441* | *3941* | *2645* | *3* | *3* | *2.108* | *55* |
| *125nM S29434 vs. 500nM S29434* | *37382* | *9805* | *27577* | *2474* | *3* | *4* | *15.77* | *55* |
| *250nM S29434 vs. 500nM S29434* | *33441* | *9805* | *23636* | *2474* | *3* | *4* | *13.51* | *55* |
|  |  |  |  |  |  |  |  |  |
| *40 min* |  |  |  |  |  |  |  |  |
| *Control vs. 62.5nM S29434* | *70084* | *53505* | *16579* | *2645* | *3* | *3* | *8.866* | *55* |
| *Control vs. 125nM S29434* | *70084* | *47936* | *22147* | *2645* | *3* | *3* | *11.84* | *55* |
| *Control vs. 250nM S29434* | *70084* | *43117* | *26967* | *2645* | *3* | *3* | *14.42* | *55* |
| *Control vs. 500nM S29434* | *70084* | *13574* | *56510* | *2474* | *3* | *4* | *32.31* | *55* |
| *62.5nM S29434 vs. 125nM S29434* | *53505* | *47936* | *5569* | *2645* | *3* | *3* | *2.978* | *55* |
| *62.5nM S29434 vs. 250nM S29434* | *53505* | *43117* | *10388* | *2645* | *3* | *3* | *5.555* | *55* |
| *62.5nM S29434 vs. 500nM S29434* | *53505* | *13574* | *39932* | *2474* | *3* | *4* | *22.83* | *55* |
| *125nM S29434 vs. 250nM S29434* | *47936* | *43117* | *4820* | *2645* | *3* | *3* | *2.577* | *55* |
| *125nM S29434 vs. 500nM S29434* | *47936* | *13574* | *34363* | *2474* | *3* | *4* | *19.64* | *55* |
| *250nM S29434 vs. 500nM S29434* | *43117* | *13574* | *29543* | *2474* | *3* | *4* | *16.89* | *55* |

| *Tukey's multiple comparisons test* | *Mean Diff.* | *95.00% CI of diff.* | *Significant?* | *Summary* | *Adjusted P Value* |  |  |  |
| --- | --- | --- | --- | --- | --- | --- | --- | --- |
|  |  |  |  |  |  |  |  |  |
| *Control* |  |  |  |  |  |  |  |  |
| *0 min vs. 10 min* | *-33794* | *-38728 to -28859* | *Yes* | ****** | *<0.0001* |  |  |  |
| *0 min vs. 20 min* | *-50647* | *-55581 to -45712* | *Yes* | ****** | *<0.0001* |  |  |  |
| *0 min vs. 30 min* | *-59356* | *-64291 to -54421* | *Yes* | ****** | *<0.0001* |  |  |  |
| *0 min vs. 40 min* | *-67905* | *-72840 to -62970* | *Yes* | ****** | *<0.0001* |  |  |  |
| *10 min vs. 20 min* | *-16853* | *-21788 to -11918* | *Yes* | ****** | *<0.0001* |  |  |  |
| *10 min vs. 30 min* | *-25562* | *-30497 to -20628* | *Yes* | ****** | *<0.0001* |  |  |  |
| *10 min vs. 40 min* | *-34111* | *-39046 to -29177* | *Yes* | ****** | *<0.0001* |  |  |  |
| *20 min vs. 30 min* | *-8709* | *-13644 to -3775* | *Yes* | ****** | *<0.0001* |  |  |  |
| *20 min vs. 40 min* | *-17258* | *-22193 to -12324* | *Yes* | ****** | *<0.0001* |  |  |  |
| *30 min vs. 40 min* | *-8549* | *-13484 to -3614* | *Yes* | ***** | *0.0001* |  |  |  |
|  |  |  |  |  |  |  |  |  |
| *62.5nM S29434* |  |  |  |  |  |  |  |  |
| *0 min vs. 10 min* | *-15291* | *-20225 to -10356* | *Yes* | ****** | *<0.0001* |  |  |  |
| *0 min vs. 20 min* | *-28853* | *-33788 to -23919* | *Yes* | ****** | *<0.0001* |  |  |  |
| *0 min vs. 30 min* | *-42723* | *-47657 to -37788* | *Yes* | ****** | *<0.0001* |  |  |  |
| *0 min vs. 40 min* | *-52772* | *-57707 to -47838* | *Yes* | ****** | *<0.0001* |  |  |  |
| *10 min vs. 20 min* | *-13563* | *-18497 to -8628* | *Yes* | ****** | *<0.0001* |  |  |  |
| *10 min vs. 30 min* | *-27432* | *-32367 to -22497* | *Yes* | ****** | *<0.0001* |  |  |  |
| *10 min vs. 40 min* | *-37482* | *-42416 to -32547* | *Yes* | ****** | *<0.0001* |  |  |  |
| *20 min vs. 30 min* | *-13869* | *-18804 to -8935* | *Yes* | ****** | *<0.0001* |  |  |  |
| *20 min vs. 40 min* | *-23919* | *-28854 to -18984* | *Yes* | ****** | *<0.0001* |  |  |  |
| *30 min vs. 40 min* | *-10050* | *-14984 to -5115* | *Yes* | ****** | *<0.0001* |  |  |  |
|  |  |  |  |  |  |  |  |  |
| *125nM S29434* |  |  |  |  |  |  |  |  |
| *0 min vs. 10 min* | *-12747* | *-17682 to -7812* | *Yes* | ****** | *<0.0001* |  |  |  |
| *0 min vs. 20 min* | *-23980* | *-28915 to -19045* | *Yes* | ****** | *<0.0001* |  |  |  |
| *0 min vs. 30 min* | *-36578* | *-41513 to -31643* | *Yes* | ****** | *<0.0001* |  |  |  |
| *0 min vs. 40 min* | *-47132* | *-52067 to -42197* | *Yes* | ****** | *<0.0001* |  |  |  |
| *10 min vs. 20 min* | *-11233* | *-16168 to -6298* | *Yes* | ****** | *<0.0001* |  |  |  |
| *10 min vs. 30 min* | *-23831* | *-28766 to -18896* | *Yes* | ****** | *<0.0001* |  |  |  |
| *10 min vs. 40 min* | *-34385* | *-39320 to -29450* | *Yes* | ****** | *<0.0001* |  |  |  |
| *20 min vs. 30 min* | *-12598* | *-17533 to -7663* | *Yes* | ****** | *<0.0001* |  |  |  |
| *20 min vs. 40 min* | *-23152* | *-28087 to -18217* | *Yes* | ****** | *<0.0001* |  |  |  |
| *30 min vs. 40 min* | *-10554* | *-15489 to -5619* | *Yes* | ****** | *<0.0001* |  |  |  |
|  |  |  |  |  |  |  |  |  |
| *250nM S29434* |  |  |  |  |  |  |  |  |
| *0 min vs. 10 min* | *-9936* | *-14870 to -5001* | *Yes* | ****** | *<0.0001* |  |  |  |
| *0 min vs. 20 min* | *-21054* | *-25989 to -16120* | *Yes* | ****** | *<0.0001* |  |  |  |
| *0 min vs. 30 min* | *-32624* | *-37559 to -27690* | *Yes* | ****** | *<0.0001* |  |  |  |
| *0 min vs. 40 min* | *-42300* | *-47235 to -37365* | *Yes* | ****** | *<0.0001* |  |  |  |
| *10 min vs. 20 min* | *-11119* | *-16053 to -6184* | *Yes* | ****** | *<0.0001* |  |  |  |
| *10 min vs. 30 min* | *-22689* | *-27623 to -17754* | *Yes* | ****** | *<0.0001* |  |  |  |
| *10 min vs. 40 min* | *-32364* | *-37299 to -27430* | *Yes* | ****** | *<0.0001* |  |  |  |
| *20 min vs. 30 min* | *-11570* | *-16505 to -6635* | *Yes* | ****** | *<0.0001* |  |  |  |
| *20 min vs. 40 min* | *-21246* | *-26180 to -16311* | *Yes* | ****** | *<0.0001* |  |  |  |
| *30 min vs. 40 min* | *-9676* | *-14610 to -4741* | *Yes* | ****** | *<0.0001* |  |  |  |
|  |  |  |  |  |  |  |  |  |
| *500nM S29434* |  |  |  |  |  |  |  |  |
| *0 min vs. 10 min* | *-3260* | *-7533 to 1014* | *No* | *ns* | *0.2104* |  |  |  |
| *0 min vs. 20 min* | *-5262* | *-9536 to -988.7* | *Yes* | **** | *0.0090* |  |  |  |
| *0 min vs. 30 min* | *-8944* | *-13218 to -4671* | *Yes* | ****** | *<0.0001* |  |  |  |
| *0 min vs. 40 min* | *-12713* | *-16986 to -8439* | *Yes* | ****** | *<0.0001* |  |  |  |
| *10 min vs. 20 min* | *-2003* | *-6276 to 2271* | *No* | *ns* | *0.6726* |  |  |  |
| *10 min vs. 30 min* | *-5685* | *-9958 to -1411* | *Yes* | **** | *0.0040* |  |  |  |
| *10 min vs. 40 min* | *-9453* | *-13727 to -5179* | *Yes* | ****** | *<0.0001* |  |  |  |
| *20 min vs. 30 min* | *-3682* | *-7956 to 591.5* | *No* | *ns* | *0.1211* |  |  |  |
| *20 min vs. 40 min* | *-7450* | *-11724 to -3177* | *Yes* | ***** | *0.0001* |  |  |  |
| *30 min vs. 40 min* | *-3768* | *-8042 to 505.3* | *No* | *ns* | *0.1073* |  |  |  |
|  |  |  |  |  |  |  |  |  |
|  |  |  |  |  |  |  |  |  |
| *Test details* | *Mean 1* | *Mean 2* | *Mean Diff.* | *SE of diff.* | *N1* | *N2* | *q* | *DF* |
|  |  |  |  |  |  |  |  |  |
| *Control* |  |  |  |  |  |  |  |  |
| *0 min vs. 10 min* | *2179* | *35972* | *-33794* | *1735* | *3* | *3* | *27.54* | *44* |
| *0 min vs. 20 min* | *2179* | *52825* | *-50647* | *1735* | *3* | *3* | *41.28* | *44* |
| *0 min vs. 30 min* | *2179* | *61535* | *-59356* | *1735* | *3* | *3* | *48.38* | *44* |
| *0 min vs. 40 min* | *2179* | *70084* | *-67905* | *1735* | *3* | *3* | *55.35* | *44* |
| *10 min vs. 20 min* | *35972* | *52825* | *-16853* | *1735* | *3* | *3* | *13.74* | *44* |
| *10 min vs. 30 min* | *35972* | *61535* | *-25562* | *1735* | *3* | *3* | *20.84* | *44* |
| *10 min vs. 40 min* | *35972* | *70084* | *-34111* | *1735* | *3* | *3* | *27.8* | *44* |
| *20 min vs. 30 min* | *52825* | *61535* | *-8709* | *1735* | *3* | *3* | *7.099* | *44* |
| *20 min vs. 40 min* | *52825* | *70084* | *-17258* | *1735* | *3* | *3* | *14.07* | *44* |
| *30 min vs. 40 min* | *61535* | *70084* | *-8549* | *1735* | *3* | *3* | *6.968* | *44* |
|  |  |  |  |  |  |  |  |  |
| *62.5nM S29434* |  |  |  |  |  |  |  |  |
| *0 min vs. 10 min* | *732.7* | *16023* | *-15291* | *1735* | *3* | *3* | *12.46* | *44* |
| *0 min vs. 20 min* | *732.7* | *29586* | *-28853* | *1735* | *3* | *3* | *23.52* | *44* |
| *0 min vs. 30 min* | *732.7* | *43455* | *-42723* | *1735* | *3* | *3* | *34.82* | *44* |
| *0 min vs. 40 min* | *732.7* | *53505* | *-52772* | *1735* | *3* | *3* | *43.01* | *44* |
| *10 min vs. 20 min* | *16023* | *29586* | *-13563* | *1735* | *3* | *3* | *11.05* | *44* |
| *10 min vs. 30 min* | *16023* | *43455* | *-27432* | *1735* | *3* | *3* | *22.36* | *44* |
| *10 min vs. 40 min* | *16023* | *53505* | *-37482* | *1735* | *3* | *3* | *30.55* | *44* |
| *20 min vs. 30 min* | *29586* | *43455* | *-13869* | *1735* | *3* | *3* | *11.3* | *44* |
| *20 min vs. 40 min* | *29586* | *53505* | *-23919* | *1735* | *3* | *3* | *19.5* | *44* |
| *30 min vs. 40 min* | *43455* | *53505* | *-10050* | *1735* | *3* | *3* | *8.191* | *44* |
|  |  |  |  |  |  |  |  |  |
| *125nM S29434* |  |  |  |  |  |  |  |  |
| *0 min vs. 10 min* | *804.3* | *13551* | *-12747* | *1735* | *3* | *3* | *10.39* | *44* |
| *0 min vs. 20 min* | *804.3* | *24784* | *-23980* | *1735* | *3* | *3* | *19.55* | *44* |
| *0 min vs. 30 min* | *804.3* | *37382* | *-36578* | *1735* | *3* | *3* | *29.81* | *44* |
| *0 min vs. 40 min* | *804.3* | *47936* | *-47132* | *1735* | *3* | *3* | *38.42* | *44* |
| *10 min vs. 20 min* | *13551* | *24784* | *-11233* | *1735* | *3* | *3* | *9.156* | *44* |
| *10 min vs. 30 min* | *13551* | *37382* | *-23831* | *1735* | *3* | *3* | *19.42* | *44* |
| *10 min vs. 40 min* | *13551* | *47936* | *-34385* | *1735* | *3* | *3* | *28.03* | *44* |
| *20 min vs. 30 min* | *24784* | *37382* | *-12598* | *1735* | *3* | *3* | *10.27* | *44* |
| *20 min vs. 40 min* | *24784* | *47936* | *-23152* | *1735* | *3* | *3* | *18.87* | *44* |
| *30 min vs. 40 min* | *37382* | *47936* | *-10554* | *1735* | *3* | *3* | *8.602* | *44* |
|  |  |  |  |  |  |  |  |  |
| *250nM S29434* |  |  |  |  |  |  |  |  |
| *0 min vs. 10 min* | *816.7* | *10752* | *-9936* | *1735* | *3* | *3* | *8.098* | *44* |
| *0 min vs. 20 min* | *816.7* | *21871* | *-21054* | *1735* | *3* | *3* | *17.16* | *44* |
| *0 min vs. 30 min* | *816.7* | *33441* | *-32624* | *1735* | *3* | *3* | *26.59* | *44* |
| *0 min vs. 40 min* | *816.7* | *43117* | *-42300* | *1735* | *3* | *3* | *34.48* | *44* |
| *10 min vs. 20 min* | *10752* | *21871* | *-11119* | *1735* | *3* | *3* | *9.063* | *44* |
| *10 min vs. 30 min* | *10752* | *33441* | *-22689* | *1735* | *3* | *3* | *18.49* | *44* |
| *10 min vs. 40 min* | *10752* | *43117* | *-32364* | *1735* | *3* | *3* | *26.38* | *44* |
| *20 min vs. 30 min* | *21871* | *33441* | *-11570* | *1735* | *3* | *3* | *9.431* | *44* |
| *20 min vs. 40 min* | *21871* | *43117* | *-21246* | *1735* | *3* | *3* | *17.32* | *44* |
| *30 min vs. 40 min* | *33441* | *43117* | *-9676* | *1735* | *3* | *3* | *7.887* | *44* |
|  |  |  |  |  |  |  |  |  |
| *500nM S29434* |  |  |  |  |  |  |  |  |
| *0 min vs. 10 min* | *861* | *4121* | *-3260* | *1503* | *4* | *4* | *3.068* | *44* |
| *0 min vs. 20 min* | *861* | *6123* | *-5262* | *1503* | *4* | *4* | *4.953* | *44* |
| *0 min vs. 30 min* | *861* | *9805* | *-8944* | *1503* | *4* | *4* | *8.418* | *44* |
| *0 min vs. 40 min* | *861* | *13574* | *-12713* | *1503* | *4* | *4* | *11.96* | *44* |
| *10 min vs. 20 min* | *4121* | *6123* | *-2003* | *1503* | *4* | *4* | *1.885* | *44* |
| *10 min vs. 30 min* | *4121* | *9805* | *-5685* | *1503* | *4* | *4* | *5.35* | *44* |
| *10 min vs. 40 min* | *4121* | *13574* | *-9453* | *1503* | *4* | *4* | *8.897* | *44* |
| *20 min vs. 30 min* | *6123* | *9805* | *-3682* | *1503* | *4* | *4* | *3.465* | *44* |
| *20 min vs. 40 min* | *6123* | *13574* | *-7450* | *1503* | *4* | *4* | *7.012* | *44* |
| *30 min vs. 40 min* | *9805* | *13574* | *-3768* | *1503* | *4* | *4* | *3.547* | *44* |
