# Supplementary figures and images for: Muscarinic-Dependent miR-182 and QR2 Expression Regulation in the Anterior Insula Enables Novel Taste Learning
Source: eNeuro. 2020 May 29;7(3):ENEURO.0067-20.2020. doi: 10.1523/ENEURO.0067-20.2020 (PMC7266141; doi:10.1523/ENEURO.0067-20.2020)

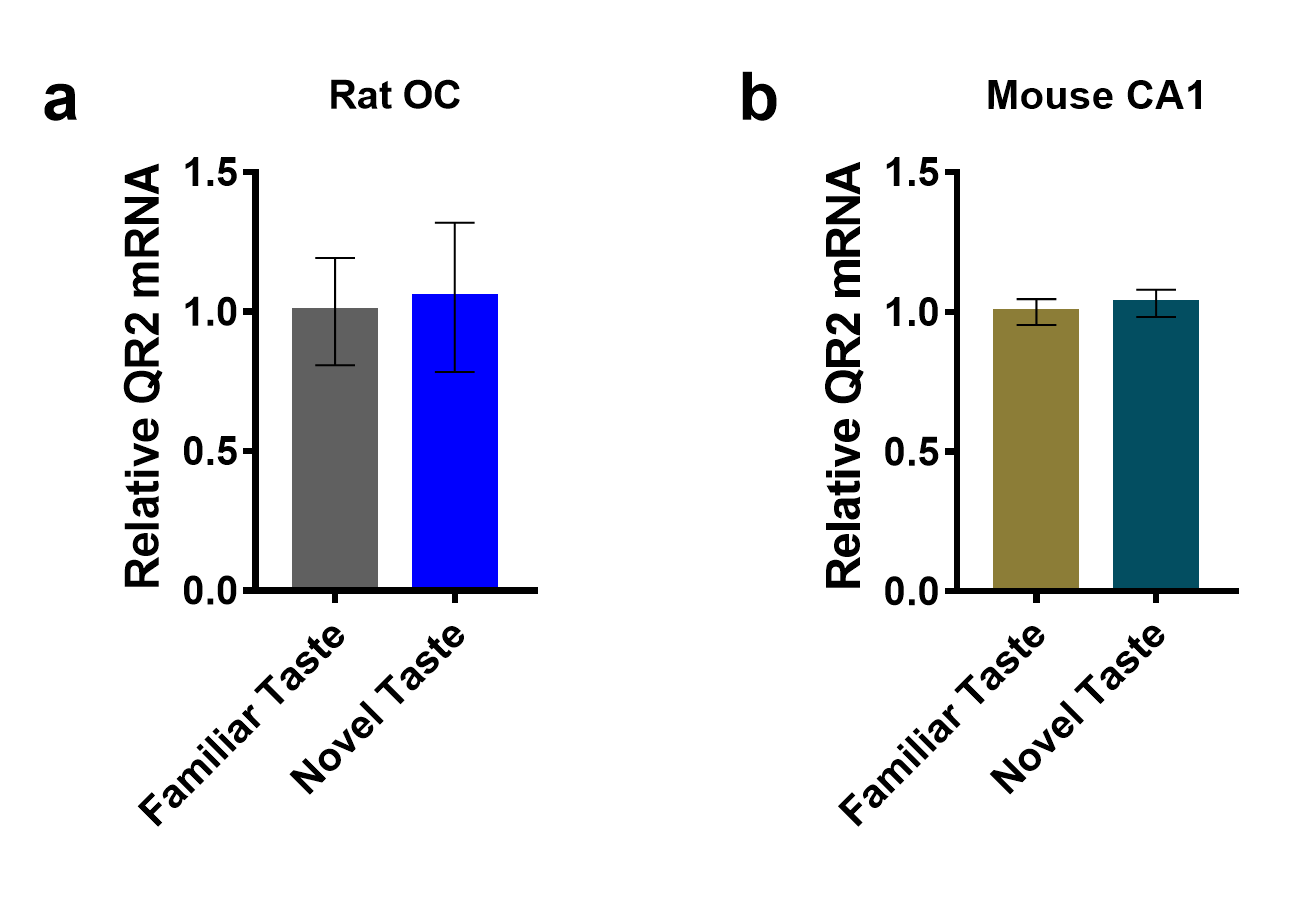

Supplement: Figure 1-1 — QR2 mRNA expression is unchanged in brain areas not associated with taste memory following novel taste consumption. a, QR2 mRNA is unchanged in rat OC following novel taste consumption. b, QR2 mRNA is unchanged in mouse CA1 following novel taste consumption. Download Figure 1-1, TIF file. [file enu-eN-NWR-0067-20-s02.tif]

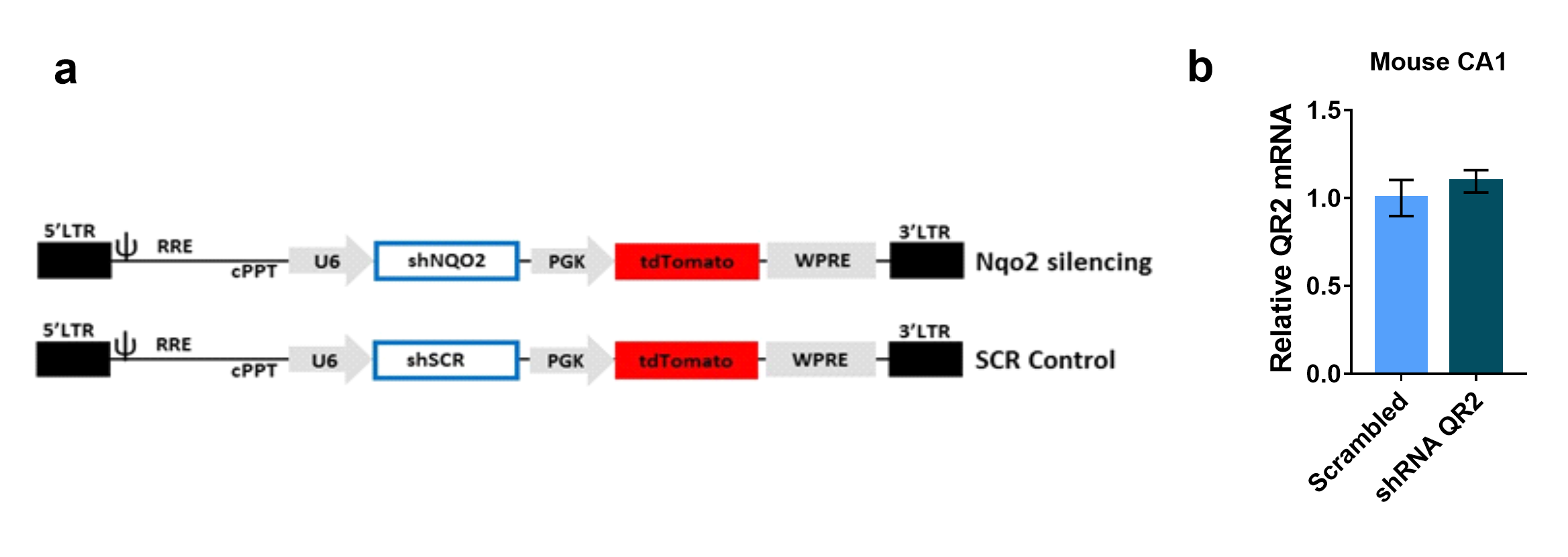

Supplement: Figure 1-2 — Lentivirus containing either shRNA targeting QR2 or a scrambled control injected to aIC did not alter QR2 mRNA expression in CA1. a, Diagram of lentivirus containing shRNA targeting QR2, or a scrambled control, used to reduce QR2 expression in mice. b, QR2 expression in CA1 remains unaffected by local aIC infection with lentivirus harboring shRNA targeting QR2. Download Figure 1-2, TIF file. [file enu-eN-NWR-0067-20-s03.tif]

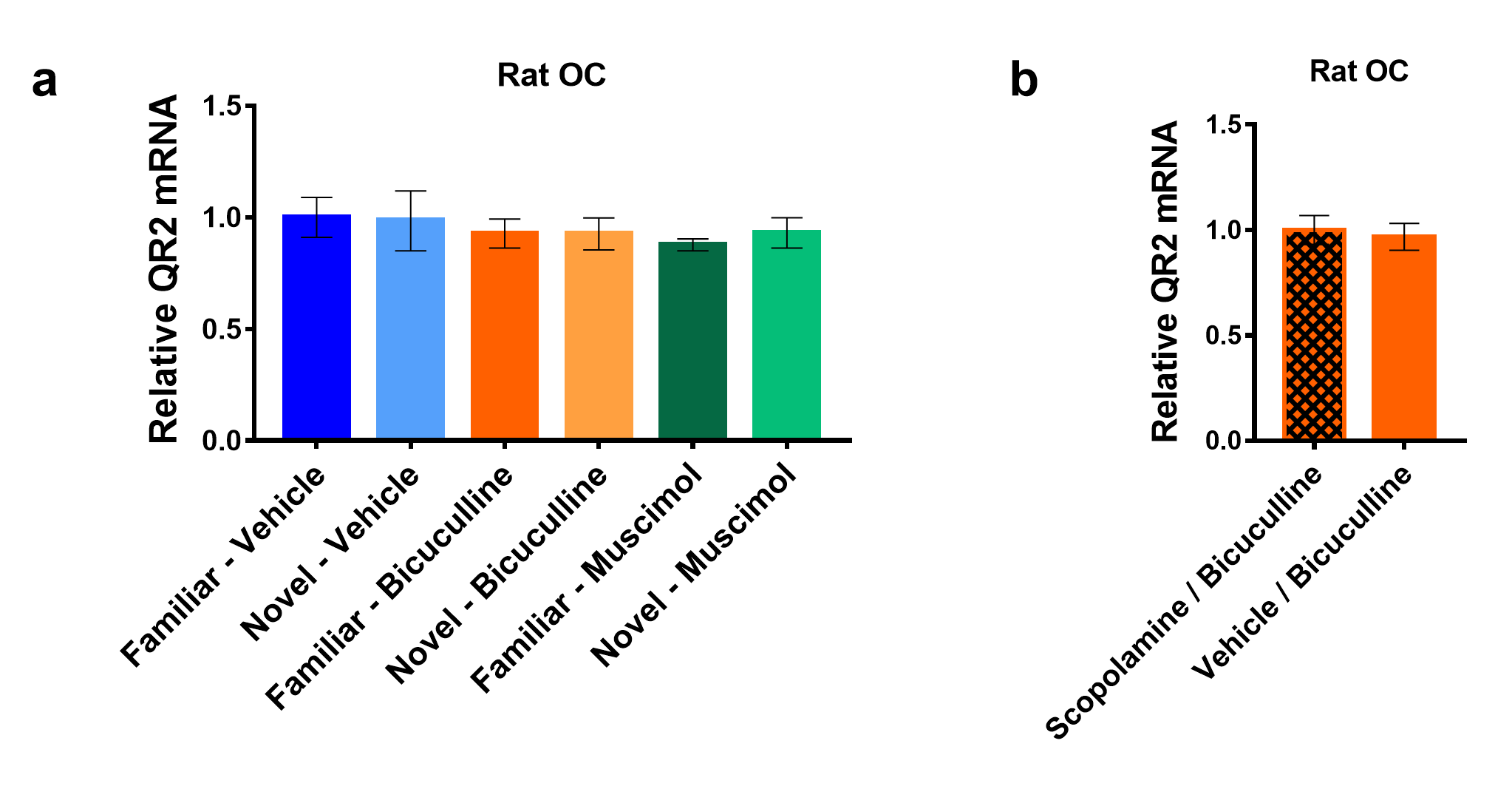

Supplement: Figure 2-1 — Local aIC GABAA receptor antagonism and scopolamine injections do not affect QR2 mRNA in the occipital cortex. a, QR2 mRNA expression is unchanged following novel taste or antagonism of GABAAR with bicuculline locally in the aIC. b, QR2 expression remains unchanged in the OC of rats, following local antagonism of GABAAR in the aIC, with or without prior injections of scopolamine. Download Figure 2-1, TIF file. [file enu-eN-NWR-0067-20-s04.tif]

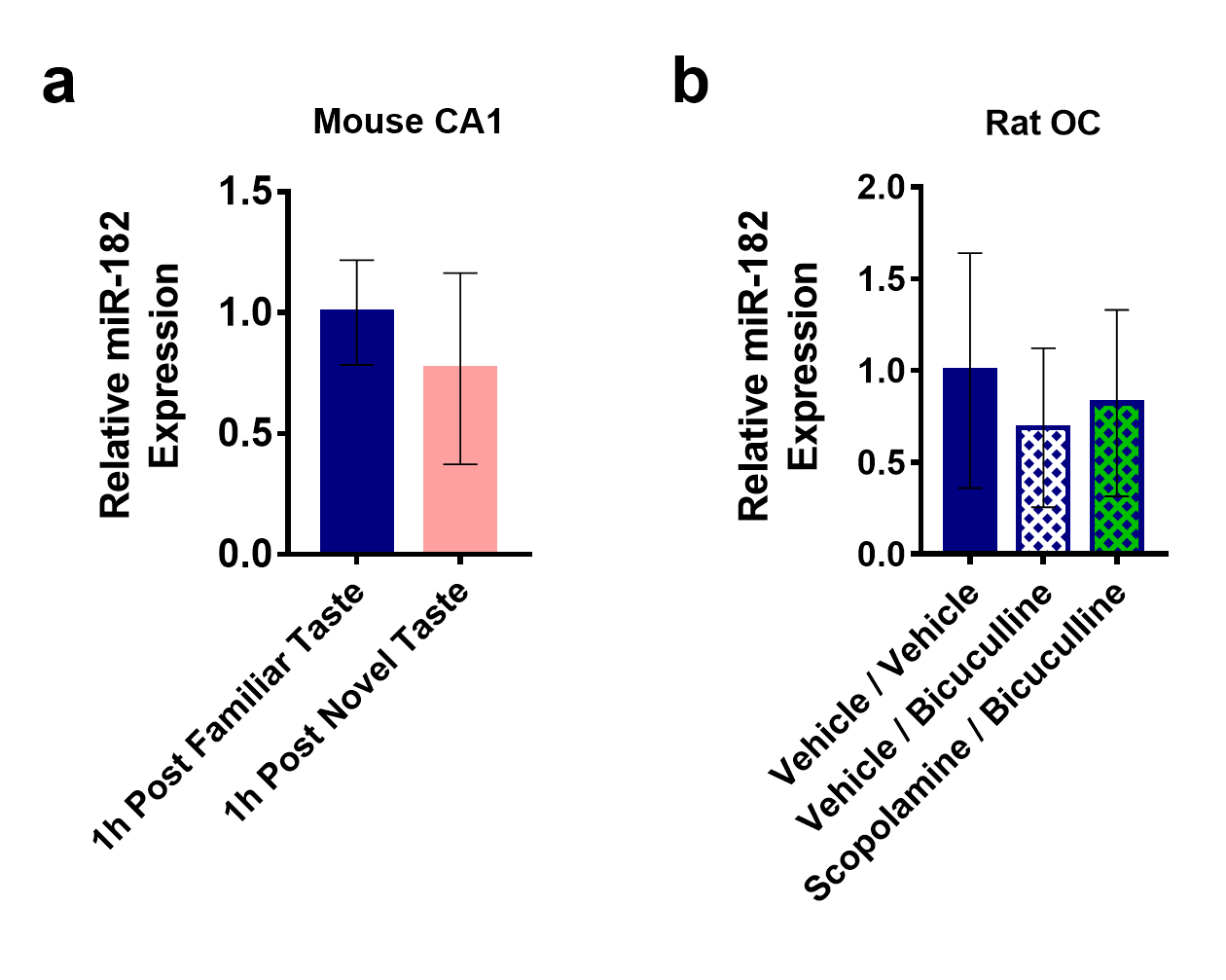

Supplement: Figure 3-1 — miR-182 expression does not increase in the mouse CA1 following novel taste consumption or in the rat OC following pharmacological manipulation locally to the rat aIC. a, miR-182 levels remain unaltered in the CA1 of mice following novel taste consumption. b, miR-182 levels remain unchanged in the OC of rats, following local aIC antagonism of GABAAR with bicuculline, with or without prior injection of scopolamine. Download Figure 3-1, TIF file. [file enu-eN-NWR-0067-20-s05.tif]

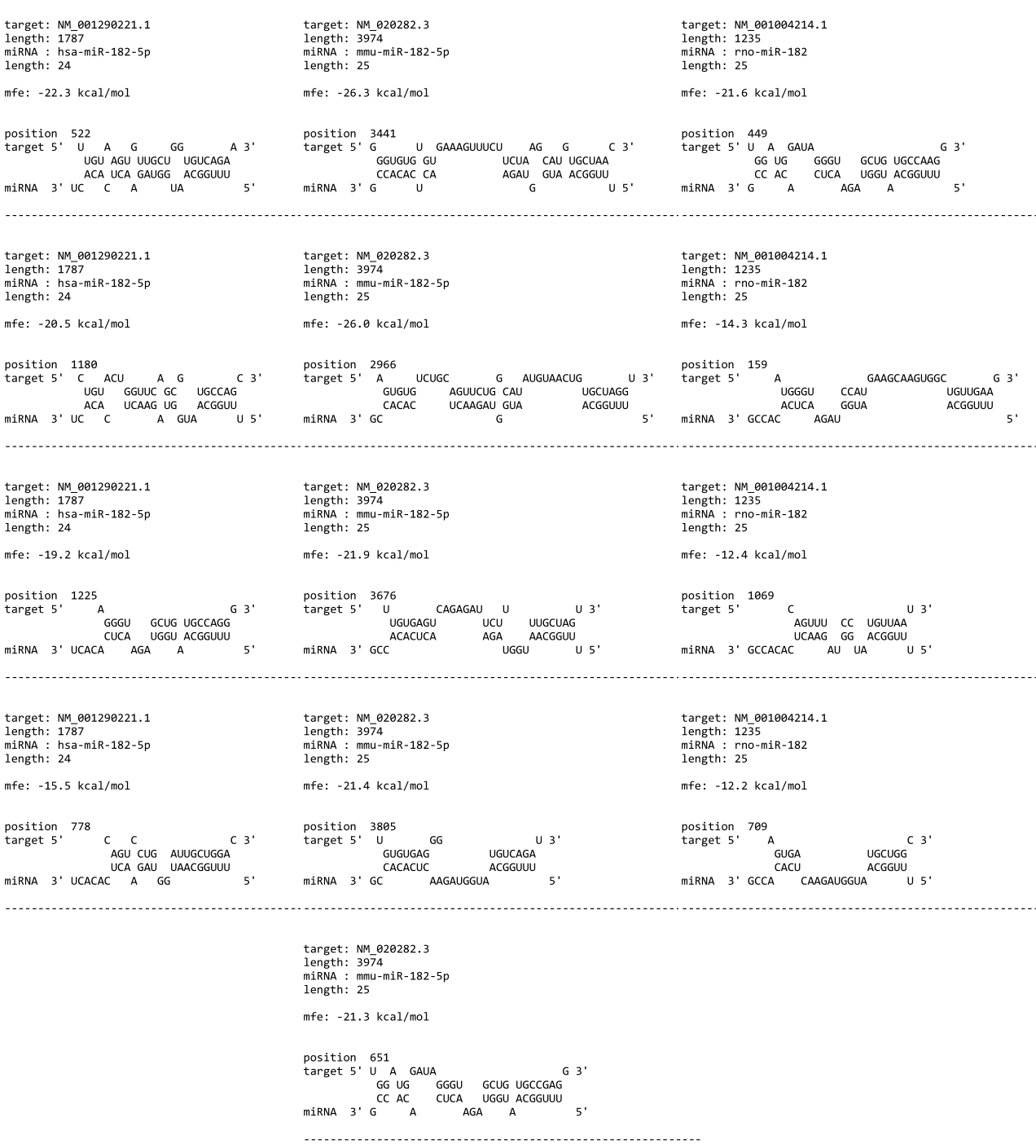

Supplement: Figure 3-2 — Predicted hybridization sites of miR-182 to QR2 mRNA in the human, mouse, and rat genome. Download Figure 3-2, TIF file. [file enu-eN-NWR-0067-20-s06.tif]

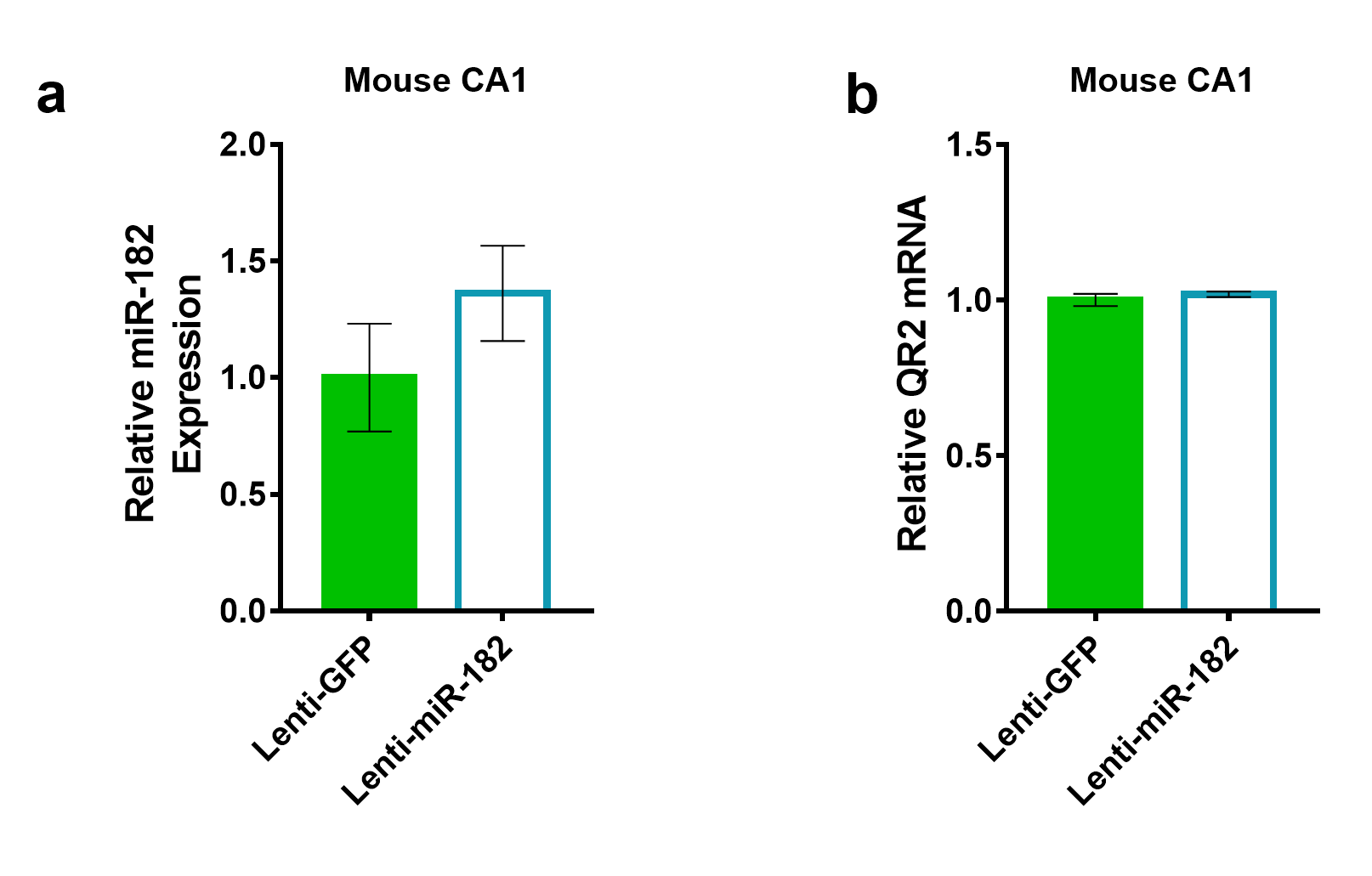

Supplement: Figure 4-1 — Mice injected in IC with a lentivirus harboring shRNA targeting QR2 do not show changes in QR2 mRNA or miR-182 expression in the hippocampus CA1. a, miR-182 levels in CA1 were not elevated following local aIC infection with a lentivirus overexpressing miR-182. b, QR2 mRNA levels in CA1 were not changed following local aIC infection with a lentivirus overexpressing miR-182. Download Figure 4-1, TIF file. [file enu-eN-NWR-0067-20-s07.tif]

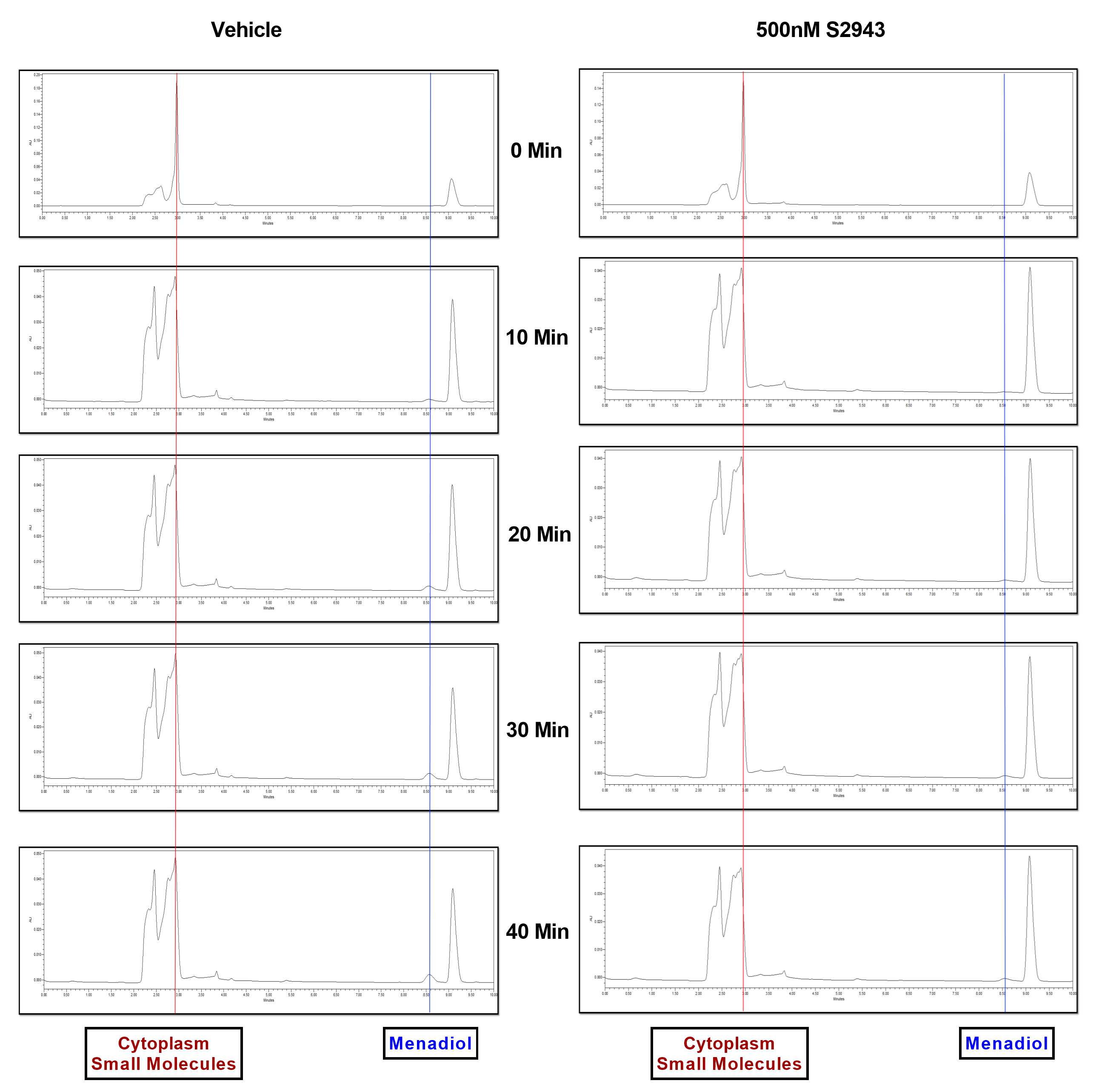

Supplement: Figure 5-1 — Time-course of menadiol formation by QR2 activity with or without S29434, using endogenous brain cytoplasmic small molecules as cofactors. Download Figure 5-1, TIF file. [file enu-eN-NWR-0067-20-s08.tif]

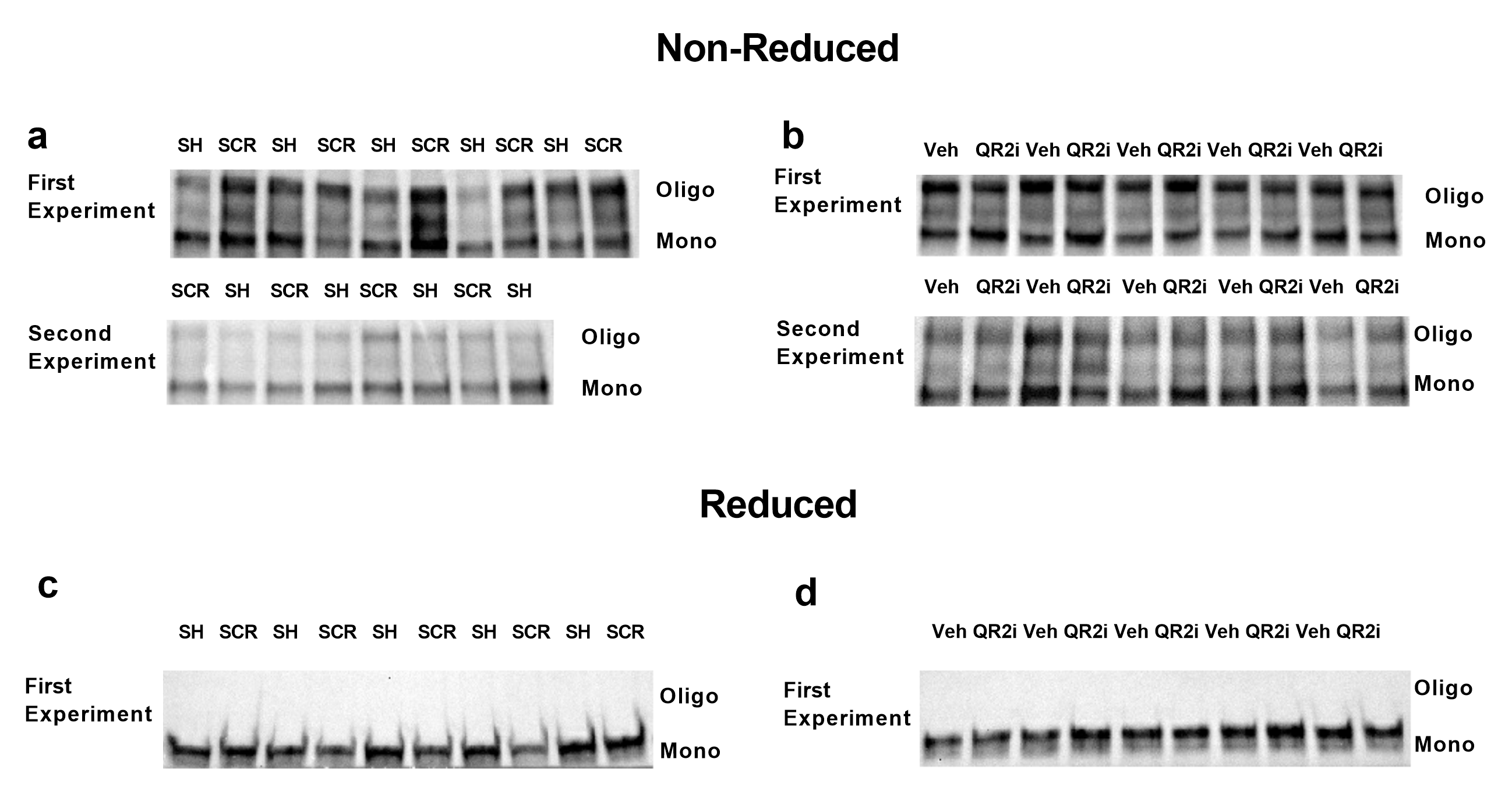

Supplement: Figure 5-2 — Cysteine redox in Kv2.1 is sensitive to ROS generated by QR2 activity. a, Kv2.1 blot following nonreducing gel electrophoresis of mouse aIC. Top, bottom, Two separate experiments in which either lentivirus containing shRNA targeting QR2 mRNA (top) or a scrambled control (bottom) was injected into the mouse aIC. b, Kv2.1 blot following nonreducing gel electrophoresis of mouse aIC samples. Top, bottom, Two separate experiments in which either QR2 inhibitor S29434 (top) or vehicle (bottom) was injected intraperitoneally. c, Kv2.1 oligomerization due to aIC redox state, as seen in a (top) is abolished in the blot following the addition of β-mercaptoethanol. d, Kv2.1 oligomerization due to aIC redox state, as seen in b (top), is abolished in the blot following the addition of β-mercaptoethanol. Download Figure 5-2, TIF file. [file enu-eN-NWR-0067-20-s09.tif]
